# Efficacy and safety of very early rehabilitation for acute ischemic stroke: a systematic review and meta-analysis

**DOI:** 10.3389/fneur.2024.1423517

**Published:** 2024-10-22

**Authors:** Ying Lou, Zhongshuo Liu, Yingxiao Ji, Jinming Cheng, Congying Zhao, Litao Li

**Affiliations:** ^1^Department of Neurology, Hebei General Hospital, Shijiazhuang, Hebei, China; ^2^Graduate School of Hebei Medical University, Shijiazhuang, Hebei, China; ^3^Hebei Provincial Key Laboratory of Cerebral Networks and Cognitive Disorders, Shijiazhuang, Hebei, China

**Keywords:** rehabilitation, early ambulation, ischemic stroke, prognosis, meta-analysis

## Abstract

**Background:**

Early rehabilitation after acute ischemic stroke (AIS) contributes to functional recovery. However, the optimal time for starting rehabilitation remains a topic of ongoing investigation. This article aims to shed light on the safety and efficacy of very early rehabilitation (VER) initiated within 48 h of stroke onset.

**Methods:**

A systematic search in PubMed, Embase, Cochrane Library, and Web of Science databases was conducted from inception to January 20, 2024. Relevant literature on VER in patients with AIS was reviewed and the data related to favorable and adverse clinical outcomes were collected for meta-analysis. Subgroup analysis was conducted at different time points, namely at discharge and at three and 12 months. Statistical analyses were performed with the help of the Meta Package in STATA Version 15.0.

**Results:**

A total of 14 randomized controlled trial (RCT) studies and 3,039 participants were included in the analysis. VER demonstrated a significant association with mortality [risk ratio (RR) = 1.27, 95% confidence interval (CI) (1.00, 1.61)], ability of daily living [weighted mean difference (WMD) = 6.90, 95% CI (0.22, 13.57)], and limb motor function [WMD = 5.02, 95% CI (1.63, 8.40)]. However, no significant difference was observed between the VER group and the control group in adverse events [RR = 0.89, 95% CI (0.79, 1.01)], severity of stroke [WMD = 0.52, 95% CI (−0.04, 1.08)], degree of disability [RR = 1.06, 95% CI (0.93, 1.20)], or recovery of walking [RR = 0.98, 95% CI (0.94, 1.03)] after stroke. Subgroup analysis revealed that VER reduced the risk of adverse events in the late stage (at three and 12 months) [RR = 0.86, 95% CI (0.74, 0.99)] and degree of disability at 12 months [RR = 1.28, 95% CI (1.03, 1.60)], and improved daily living ability at 3 months [WMD = 4.26, 95% CI (0.17, 8.35)], while increasing severity of stroke during hospitalization [WMD = 0.81, 95% CI (0.01, 1.61)].

**Conclusion:**

VER improves activities of daily living (ADLs) and lowers the incidence of long-term complications in stroke survivors. However, premature or overly intense rehabilitation may increase mortality in patients with AIS during the acute phase. PROSPERO registration number: CRD42024508180.

**Systematic review registration:**

This systematic review was registered with PROSPERO (https://www.crd.york.ac.uk/PROSPERO/). PROSPERO registration number: CRD42024508180.

## 1 Introduction

Acute ischemic stroke (AIS) refers to the abrupt onset of focal neurological dysfunction resulting from insufficient blood supply to the brain or determined according to objective evidence of vascular origin observed through imaging or pathological examination (1). It features high incidence, recurrence, disability, and mortality worldwide (2), and represents approximately 80% of all stroke cases (3). In the Trial of Org 10,172 in Acute Stroke Treatment (TOAST) classification system, large-artery atherosclerosis and cardioembolism are the main etiologies of stroke, with contributing risk factors including cardiovascular, endocrine, and others. Stroke, as the second leading cause of death and disability worldwide according to the Global Burden of Disease Study in 2016 (4), imposes substantial health and economic burdens in both developed and developing nations. Moreover, there has been a gradual increase in stroke incidence among young populations (5, 6). The progression of ischemic stroke is commonly categorized into acute, subacute, and chronic phases; however, the temporal boundaries of these stages are inconsistently defined. In the present study, acute stroke was defined as a stroke that occurs within 7 days after the onset, subacute stroke was a stroke occurring more than 7 days and less than 3 months after the onset, and chronic stroke generally referred to a non-recurrent stroke that lasts 3 months. Despite advancements in stroke unit management and early revascularization which promote timely recovery of brain blood flow in recent years, 50% of patients became chronically disabled with low life quality (7), because neural restoration was constrained by a narrow therapeutic window and irreversible damage to neuron. Some stroke survivors experience lingering complications and sequelae, particularly motor impairment and cognitive decline (8). In a recent study, it was demonstrated that acute or subacute stroke patients with *Clostridium difficile* infection exhibited significant improvement in basic living ability at discharge after 3 h of daily neurorehabilitation, but no significant difference was found in comparison to non-infected patients (9). Therefore, in addition to standard care, systematic, regular and intensive rehabilitation is of great importance in the early period of stroke even in the presence of other complications such as infections, unless patients have malaise or worse symptoms.

Post-stroke rehabilitation, as a long and relatively safe intervention, is conducive to restoring limb motivation, improving walking and balancing abilities, and reducing the incidence of disability, falls and cardiorespiratory diseases (10). Initiating rehabilitation promptly after the stabilization of vital signs would help to accelerate the recovery of central nervous system and prevent potential complications (11). Sun et al. suggested that early rehabilitation could influence the expression of serum inflammatory factors, such as vascular endothelial growth factor (VEGF), tumor necrosis factor-α (TNF-α), interleukin-10, and stromal cell-derived factor-1α, and motivate endothelial progenitor cells (12), thereby promoting endothelial formation and vascular regeneration in AIS (13). However, the optimal timing for commencing early rehabilitation after stroke remains controversial, with uncertainty regarding the safety and efficacy of very early rehabilitation (VER) in patients with AIS. Firstly, for patients with post-stroke paralysis, very early out-of-bed activities may precipitate falls due to weak limb strength or poor balancing ability. Moreover, significant head position change after stroke would decrease cerebral blood flow (14), which could aggravate ischemia in the infarct area and lead to deterioration of the disease, while maintaining a supine position could increase cerebral perfusion pressure and boost collateral circulation to support the ischemic penumbra (15, 16). Despite the absence of definitive evidence and a lack of consensus regarding the optimal rehabilitation strategy, which involves starting time, frequency and intensity (17, 18), VER has been advocated within some published stroke guidelines (19, 20), and merits further exploration. Notably, a recent meta-analysis of randomized controlled trials (RCTs) conducted in 2021 revealed positive efficacy of early rehabilitation at 3 months. No statistical difference in adverse events and disability rate was noted between the VER group and control group, but the study did not assess outcomes in different endpoints (21).

This meta-analysis included RCTs to evaluate the effects of initiating VER within 48 h of stroke onset on short- and long-term recovery. Additionally, a subgroup analysis at different time points (at discharge, 3 months and 12 months) was performed to observe the dynamic changes of the efficacy and safety of VER, which could serve as a reference for clinical practice.

## 2 Methods

This systematic review was conducted and reported according to the guidelines of the Preferred Reporting Items for Systematic Reviews and Meta-Analyses (PRISMA) (22), and registered with PROSPERO (CRD42024508180).

### 2.1 Search strategy

Two investigators independently searched PubMed, Embase, Web of Science, and the Cochrane Library from inception until January 20, 2024. Medical Subject Headings (MeSH) terms and free-text words, including “ischemic stroke” and “early ambulation or early mobilization or rehabilitation” and “early,” were employed in the search process. Other relevant literature was acquired based on the reference list of included studies. The search terms and strategies are detailed in Supplementary material 1.

### 2.2 Inclusion and exclusion criteria

The inclusion criteria of the meta-analysis were as follows: (1) Population: Patients aged 18 or older who met the diagnostic criteria for AIS and were admitted to the hospital within 48 h of the onset were included; (2) Intervention: VER was initiated within 48 h of stroke onset in the experiment group; (3) Control: The control group received either standard care or delayed rehabilitation; (4) Outcomes: The main outcomes included indicators of safety, severity and function. Safety and severity indicators included: (1) Mortality: It was defined as the proportion of death or modified Ranking Score (mRS) rated as 6. The mRS was an ordinal scale ranging from 0 (no disability) to 5 (severe disability), with a score of 6 indicating decease (23). (2) Adverse events: It was defined as the proportion of non-fatal adverse events after stroke, including immobility-related and neurological complications after the attack. (3) National Institutes of Health Stroke Scale (NIHSS): It was the most widely employed measure of stroke severity to evaluate the efficacy of treatment strategies for AIS (24). Indicators of physical function encompassed: (1) mRS: It was a favorable outcome defined as mRS of 0–2 (no or minimum disability), and the proportion of mRS of 0–2 was used to assess the improvement of disability. (2) Barthel Index (BI): It was a valid 10-item tool used to assess patient independence for ADLs in stroke (25). There were two kinds of scales, with a total of score of 0–100 and 0–20, respectively (26, 27). The two scales had consistent content and clinimetrics. The total score summed to 20 or 100, with higher scores indicating better performance (26). Among the eligible studies, Cumming and Langhorne applied the 0–20 scale, while the others employed the 0–100 scale. (3) Fugl-Meyer assessment (FMA): FMA consisted of two subscales to assess upper and lower extremities motor function. Higher scores mean better limb mobility (28). (4) The proportion of walking 50 m unassisted: It referred to the proportion of patients walking over 50 m by 3 months without assistance and was adopted to estimate independent walking ability. (5) Study design: All studies were RCTs and published in English. Studies were excluded if the full text was not accessible, the details regarding intervention was unclear, the intervention did not meet the criteria, or relevant outcome indicators were absent. Besides, reviews, meta-analyses, case reports, conference abstracts, incomplete clinical protocols, animal experiments and duplicate publications were also removed.

### 2.3 Data extraction

Two researchers independently screened the literature based on the inclusion and exclusion criteria, extracted important information from every eligible study, and developed standardized tables. The extracted data included the following variables: the first author, publication year, country, sample size, average age, sex ratio, intervention of each group, the start time of early rehabilitation in the VER group and outcome measures. Discrepancies were resolved through discussion or consultation with another author to achieve consensus.

### 2.4 Quality assessment of included studies

The quality of included studies was assessed using the Cochrane Collaboration’s tool. The assessment for possible bias identification included seven items: generation of randomized sequences, allocation concealment blinding of implementers and participants, blinding of outcome assessors, completeness of outcome data, selective reporting of study results, and other potential sources of bias (29, 30). The studies were classified as having low, unclear, or high risk of bias. Those meeting all criteria were categorized as having a “low risk,” which indicated high quality and minimal overall bias. Those partially meeting the criteria were classified as having an “unclear risk,” which suggested moderate potential for bias. Those failing to meet the criteria were labeled as having a “high risk,” which reflected a notable risk of bias and lower quality. Consensus was reached on any discrepancies or disagreements.

### 2.5 Statistical analysis

Stata 15.0 software was used to perform statistical analysis of the collected data. Weighted mean difference (WMD) and 95% confidence interval (CI) were utilized to describe continuous variables, and relative risk (RR) and 95% CI were used for dichotomous variables. Different statistical models were chosen according to the presence of heterogeneity, which was assessed by *I*^2^ values or Cochran’s Q-statistics. Values of *I*^2^ < 25%, ≥25 to <50%, ≥50 to <75%, and ≥75% indicated none, low, moderate, and high heterogeneity, respectively. Heterogeneity was considered significant when *p* < 0.05 and *I*^2^ > 50%. The data were analyzed through a fixed-effects model when the *I*^2^ value was less than 50% (*p* > 0.05, *I*^2^ < 50%). Instead, if the *I*^2^ value was equal to or greater than 50% (*p* < 0.05, *I*^2^ ≥ 50%), a sensitivity analysis was conducted to explore potential sources of heterogeneity, and statistical analysis was performed with a fixed-effects model. In addition, publication bias was evaluated via a funnel plot and was quantified through Egger tests. Finally, subgroup analysis by time was conducted to further understand the effects of the intervention on outcomes such as adverse events, mRS, BI, and NIHSS in both short and long terms.

## 3 Results

### 3.1 Study selection

**Figure 1:**
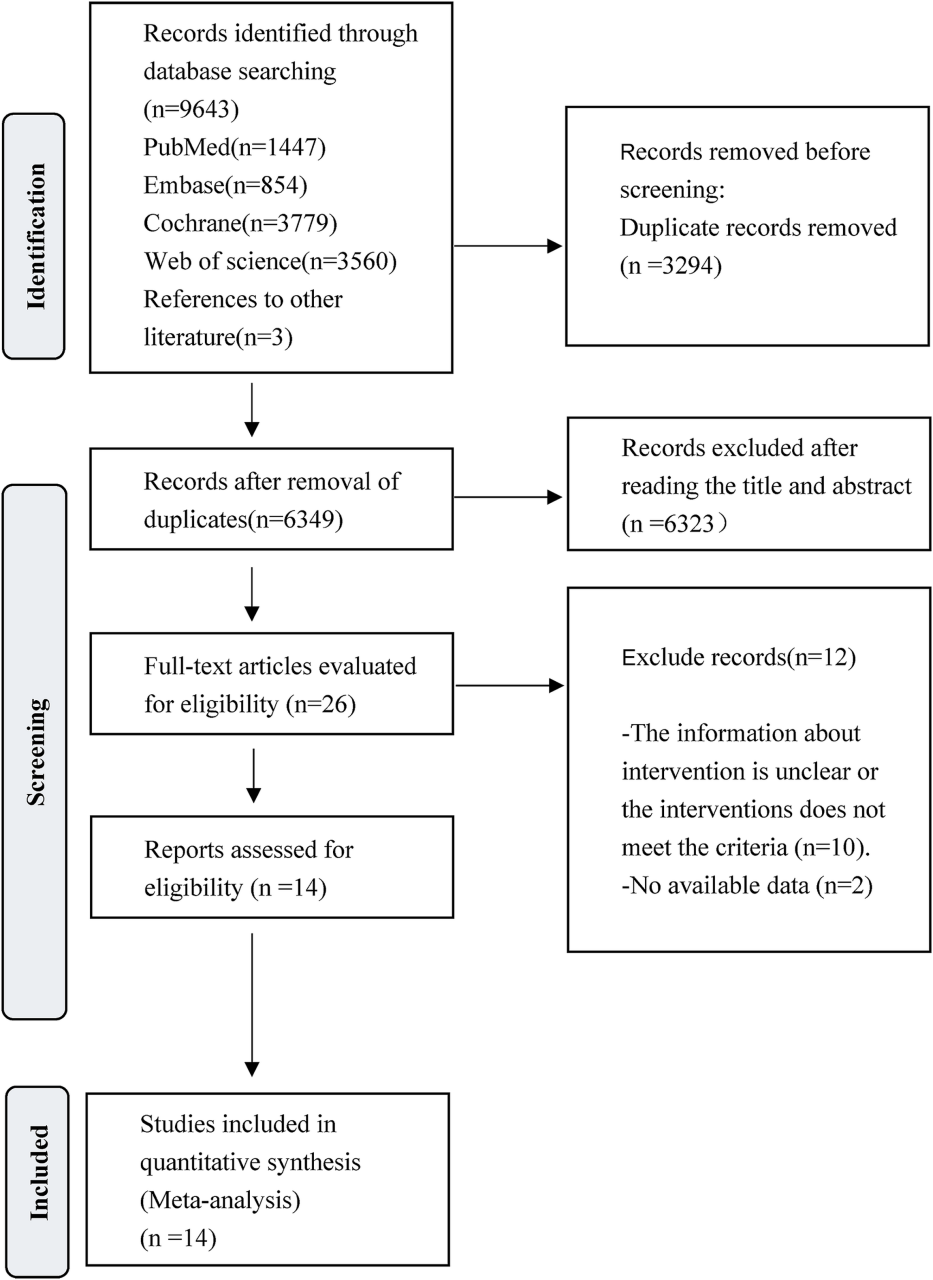
PRISMA flow diagram of the study process. Preferred Reporting Items for Systematic Reviews and Meta-Analyses (PRISMA).

The literature search and selection process is presented in Figure 1. A total of 9,643 studies were initially identified for screening. After the removal of 3,294 duplicate articles and 6,323 articles that did not meet the predefined inclusion criteria, the full texts of 26 RCTs were reviewed. Following this full-text assessment, two studies were excluded due to insufficient data for statistical analysis. Further scrutiny revealed that 10 were ineligible because they either initiated VER on the experiment group after 48 h, commenced rehabilitation on the control group after 24 h but within 48 h, or lacked specification regarding the timing of interventions. At last, a total of 14 studies met the eligibility criteria and were finally included for subsequent analysis. Among them, Van Wijk et al. and Cumming et al. reported extended outcomes of the same study population in the research by Bernhardt et al. in 2007. Bernhardt et al. provided additional results in a study published in 2021 to complement the 2015 research.

### 3.2 Characteristics of trials and risks of bias

**Figure 2:**
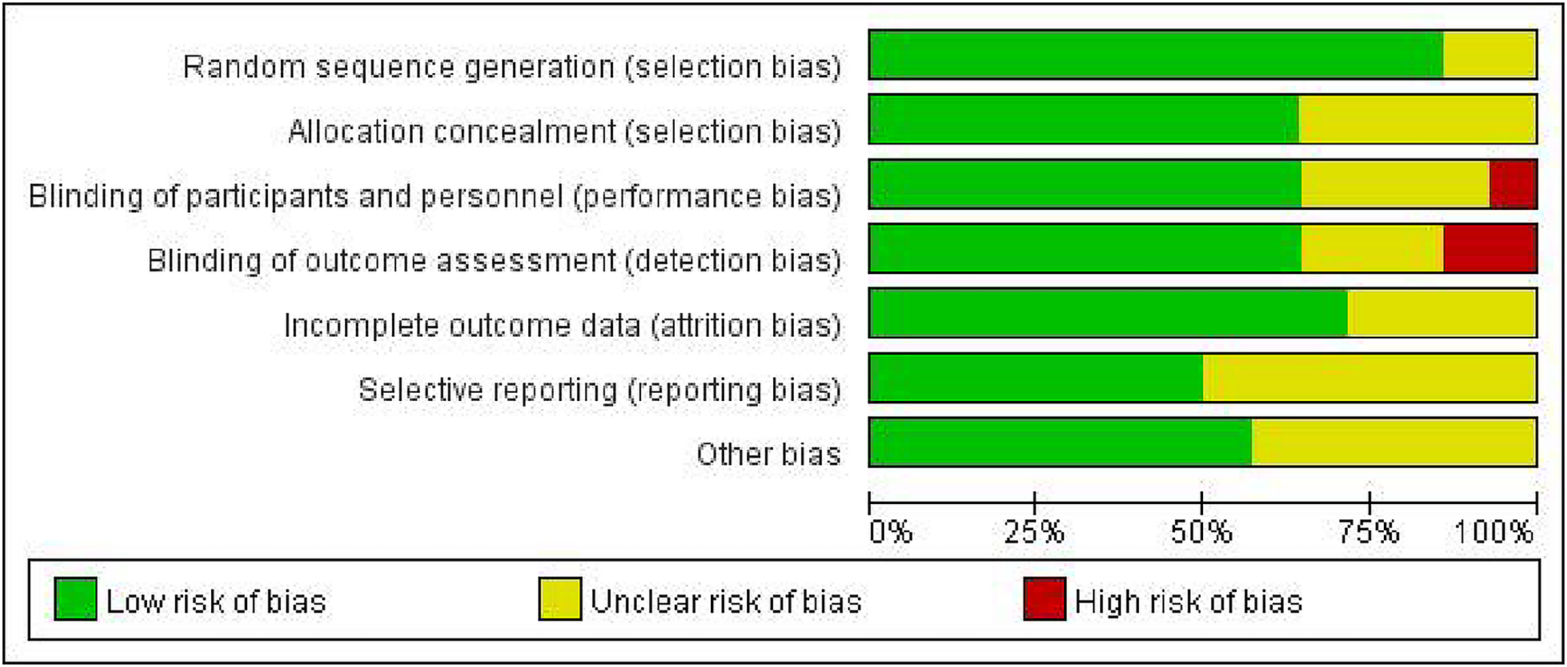
Graph for risk of bias.

**Figure 3:**
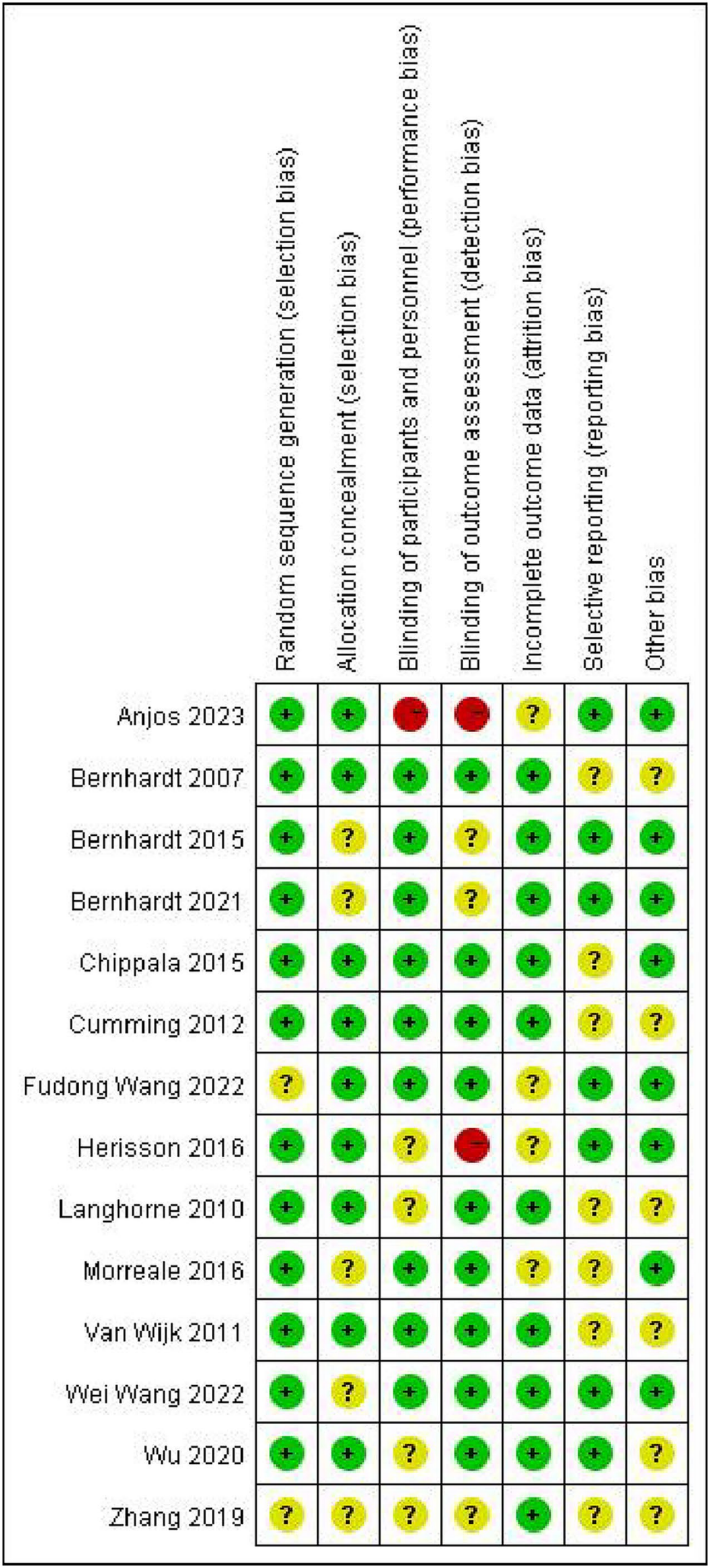
Summary for risk of bias.

The characteristics of 14 eligible RCTs are detailed in Table 1. The total sample size comprised 3,039 participants, with 1,544 in the experiment group and 1,495 in the control group. The study populations were from diverse geographic regions: four studies originated from China (31–34), five from Australia (35–39), one from India (40) and four from Europe (41–44). Regarding the time of initiating rehabilitation, VER was started within 24 h after stroke in 10 studies (17, 35–37, 39–44), between 24 and 48 h in two studies (32, 33) and within 48 h in two studies (31, 34). The risks of bias are shown in Figures 2, 3.

**Table 1:** Characteristics of trials.

Study	Year	Country	Sample size		Gender (M/F)	Mean age (years)		Intervention		Start-up time of EG	Outcome (Assessment time)
			EG	CG		EG	CG	EG	CG		
Bernhardt	2007	Australia	38	33	38/33	74.6	74.9	VER (very early out-of-bed activities) + SC	SC	≤24 h	F5 (at 3 months); F7 (at 3 months; at 12 months)
Langhorne	2010	U.K.	16	16	16/16	63	71	VER (very early out-of-bed activities) + SC	SC	≤24 h	F3; F5; F6; F7 (at 3 months)
Van Wijk (Further results of Bernhardt 2007)	2011	Australia	38	33	38/33	74.6	74.9	VER (very early out-of-bed activities) + SC	SC	≤24 h	F6 (at 3 months)
Cumming (Further results of Bernhardt 2007)	2012	Australia	38	33	38/33	74.6	74.9	VER (very early out-of-bed activities) + SC	SC	≤24 h	F3 (at 3 months; at 12 months)
Bernhardt	2015	Australia	1,054	1,050	1,286/818	72.3	72.7	VER (very early out-of-bed activities) + SC	SC	≤24 h	F2; F5; F6; F7 (at 3 months)
Chippala	2015	India	40	40	42/38	59.32	60.57	VER (very early out-of-bed activities) + SC	SC	≤24 h	F3 (at 3 months)
Herisson	2016	France	63	75	89/49	68.1	71.2	Very early sitting + SC	Progressive sitting at different angles in the first few days + SC Day0 at 30° Day1 at 45° Day2 at 60°	≤24 h	F3 (at 3 months); F4 (at discharge; at 3 months); F5 (at 3 months); F6 (during hospitalization); F7 (at discharge; at 3 months)
Morreale	2016	Italy	110	60	122/48	64	63	VER (very early out-of-bed activities) + SC	DR (intensive rehabilitation from the 5th day)	≤24 h	F3 (at 3 months; at 12 months); F6 (at 3 months)
Zhang	2019	China	48	48	57/39	Age group ≥40, 21 <40, 27	≥40, 20 <40, 28	VER (limb movement)	SC	24–48 h	F1 (unmentioned)
Wu	2020	China	16	15	22/9	61.06	62.67	VER (lower limb exercise strengthening)	SC	≤24–48 h	F2; F7 (at 3 months)
Bernhardt (Further result of Bernhardt 2015)	2021	Australia	1,054	1,050	1,286/818	72.3	72.7	VER (very early out-of-bed activities) + SC	SC	≤24 h	F5; F6 (at 14 days)
Fudong Wang	2022	China	56	54	65/45	60.27	61.04	VER (limb movement in or out of bed) + SC	DR (within 72–96 h) + SC	24–48 h	F1; F7 (at 3 months)
Wei Wang	2022	China	52	51	82/21	58	62	VER (very early out-of-bed activities) + Routine rehabilitation	Routine rehabilitation (≥48 h)	≤48 h	F3; F5; F6; F7 (at 3 months; at 12 months)
Anjos	2023	Germany	51	53	55/49	61.80	58.89	VER (very early out-of-bed activities)	SC	≤12 h	F4; F7 (at discharge; at 3 months)

M, male; F, female; EG, Experimental Group; CG, Control Group; VER, Very early rehabilitation; SC, standard cure; DR, delayed rehabilitation; F1, FMA, Fugl-Meyer Assessment, measuring motor impairment; F2: Proportion of walking 50m unassisted; F3, BI, Barthel Index; F4, NIHSS, the National Institutes of Health Stroke Scale; F5, death/mortality; F6, the proportion of post-stroke adverse events; F7, mRS, modified Rankin Scale.

### 3.3 Meta-analysis results

#### 3.3.1 Mortality

**Figure 4:**
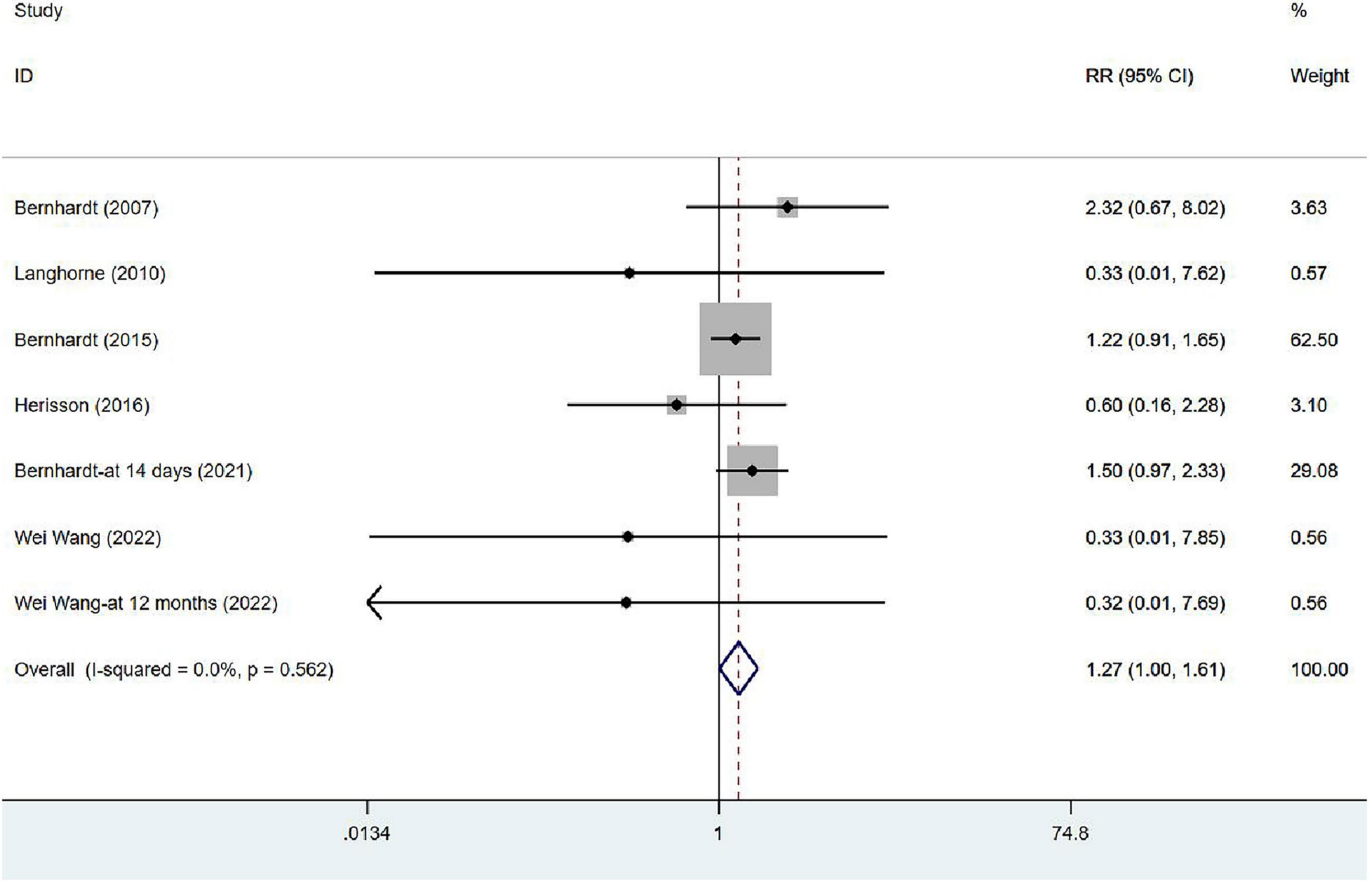
Forest plot of meta-analysis of mortality. Comparison: the effect of VER on the mortality rate after stroke. Statistical method: Inverse-variance of the fixed effects model [relative risk (RR) and 95% confidence interval (CI)]. very early rehabilitation (VER).

In six studies (17, 34, 35, 39, 41, 43), mortality was assessed at varying intervals following stroke onset: five studies at 3 months, one at 14 days, and one at 12 months. Unless otherwise specified, all figures represent assessments at 3 months after the onset. Given the absence of heterogeneity (*I*^2^ = 0.0%, *p* = 0.562), a fixed-effects model was employed to analyze the mortality data. The results of the meta-analysis (Figure 4) suggested that VER would raise the risk of death in stroke patients [RR = 1.27, 95% CI (1.00, 1.61)].

#### 3.3.2 Adverse events and its subgroup analysis

**Figure 5:**
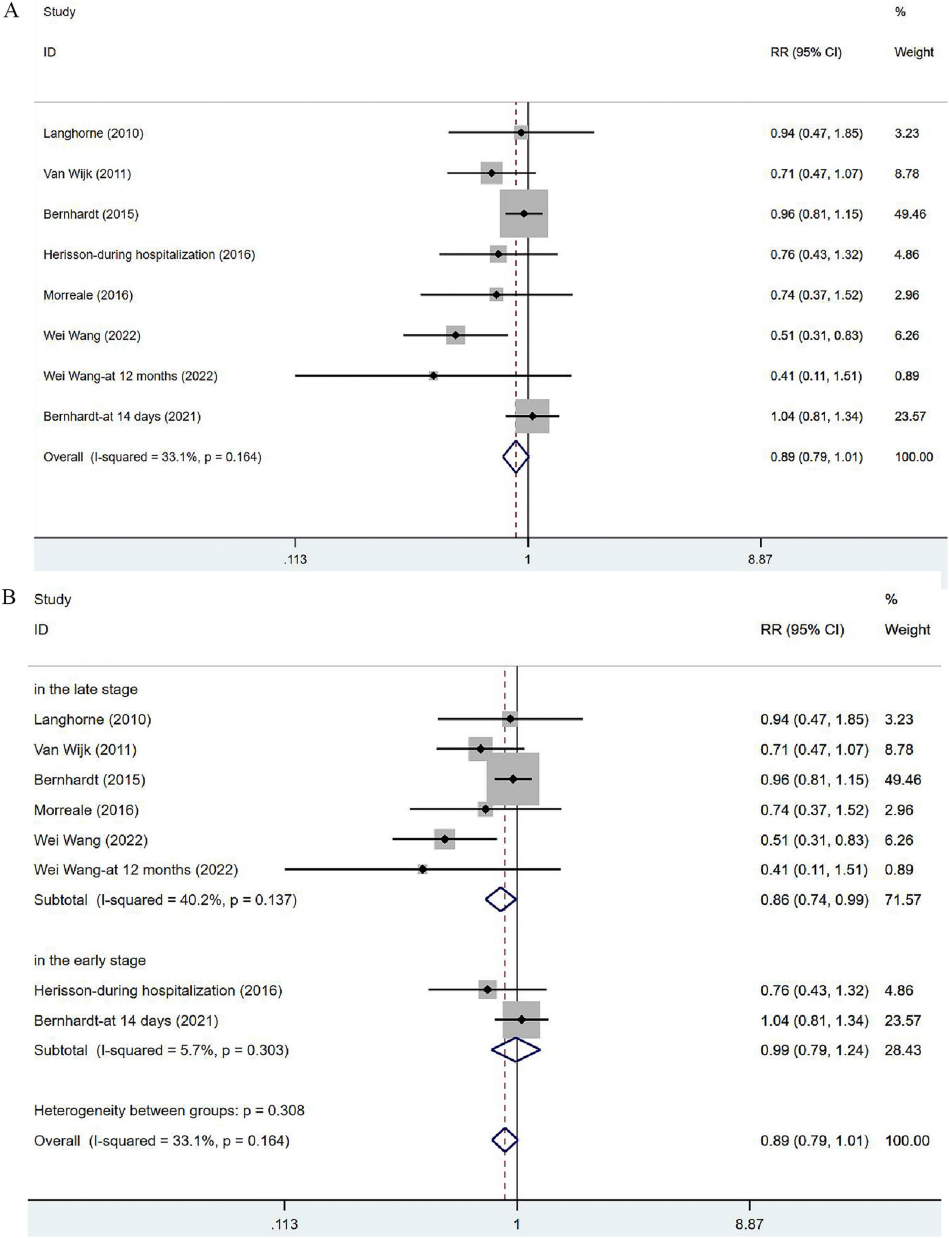
Forest plot of the adverse events. (A) Forest maps of meta-analysis of adverse events. (B) Forest maps of subgroup analysis of adverse events. (A) Comparison: the effect of VER on the proportion of adverse events after stroke. Statistical method: Inverse-variance of the fixed effects model [relative risk (RR) and 95% confidence interval (CI)]. Very early rehabilitation (VER). (B) Comparison: the effect of VER on the proportion of adverse events after stroke at different endpoints. Very early rehabilitation (VER); RR, risk ration; 95% CI, 95% confidence interval.

Seven articles (34, 35, 37, 39, 41–43) involved the non-fatal adverse events. In view of the low heterogeneity (*I*^2^ = 33.1%, *p* = 0.164), a fixed-effects model was used. The results of the analysis (Figure 5A) demonstrated no significant difference in incidence of adverse events between the VER group and the control group [RR = 0.89, 95% CI (0.79, 1.01)]. Two of these studies evaluated the incidence of complications at 14 days after stroke or during hospitalization, in the early stage of stroke recovery, while the remaining five focused on the recovery period after stroke (at 3 months or 12 months), reflecting a relatively long-term outcome. Subgroup analysis (Figure 5B) based on time was performed to dynamically observe the short-term or long-term effects of VER. VER could decrease the risk of adverse events assessed at three and 12 months [RR = 0.86, 95% CI (0.74, 0.99)], but could not significantly lower the risk in the early stage [RR = 0.99, 95% CI (0.79, 1.24)].

#### 3.3.3 Severity of stroke: NIHSS and its subgroup analysis

**Figure 6:**
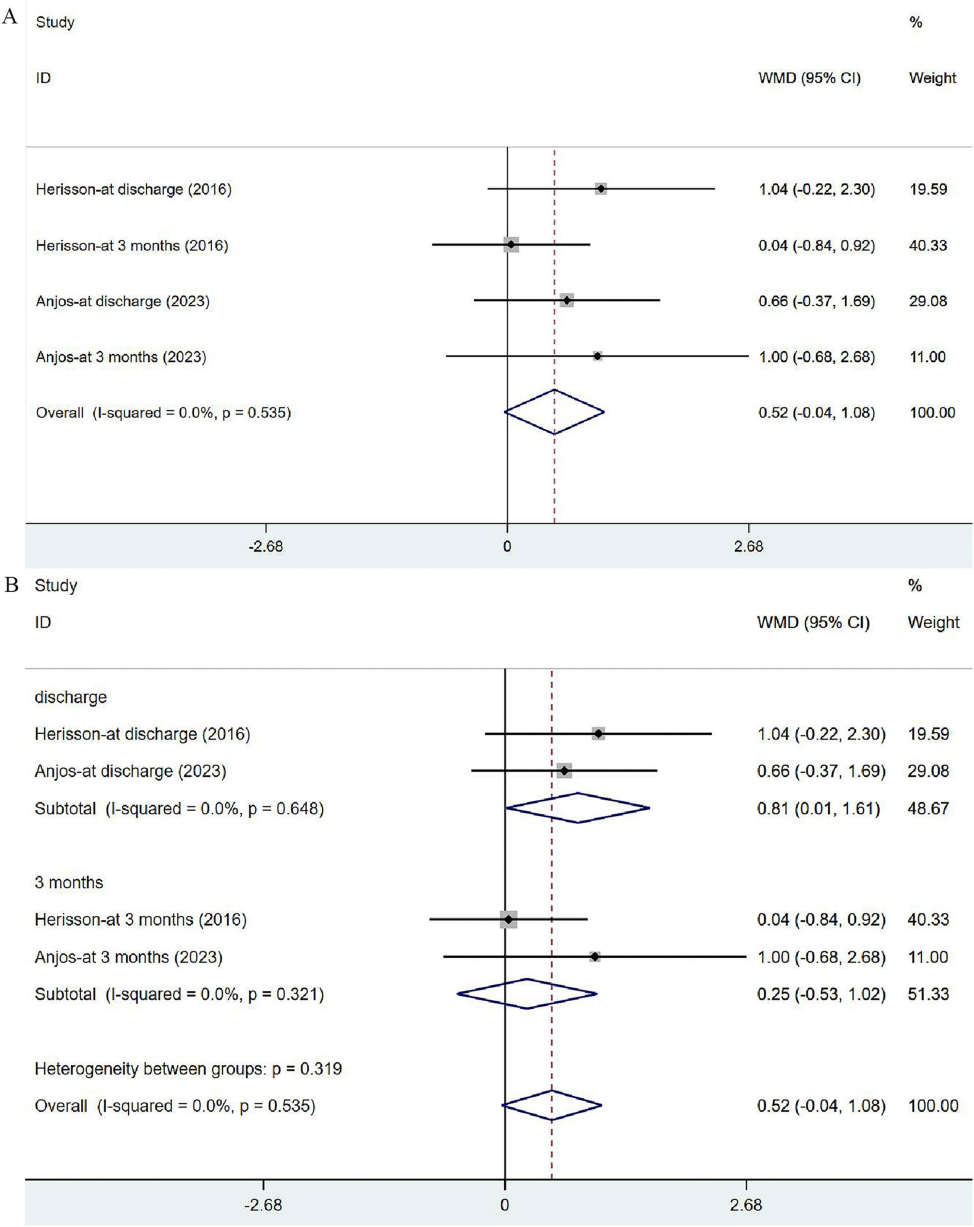
Forest plot of the NIHSS. (A) Forest maps of meta-analysis of NIHSS. (B) Forest maps of subgroup analysis of NIHSS. (A) Comparison: the effect of VER on NHISS. Statistical method: Inverse-variance of the fixed effects model [weighted mean difference (WMD) and 95% confidence interval (CI)]. National Institutes of Health Stroke Scale (NIHSS); very early rehabilitation (VER). (B) Comparison: the effect of VER on NIHSS after stroke at different endpoints. National Institutes of Health Stroke Scale (NIHSS); very early rehabilitation (VER); weighted mean difference (WMD); 95% CI: 95% confidence interval.

Two studies (41, 44) reported the outcomes of NIHSS at discharge or at 3 months. The heterogeneity was not significant (*I*^2^ = 0.0%, *p* = 0.535). The fixed-effects model was applied (Figure 6A), and the analysis results revealed no significance in stroke severity between the two groups [WMD = 0.52, 95% CI (−0.04, 1.08)]. In the subgroup analysis (Figure 6B), there was a slight difference in NHISS assessed at discharge between the two groups [WMD = 0.81, 95% CI (0.01, 1.61)] but no significant difference was observed at 3 months [WMD = 0.25, 95% CI (−0.53, 1.02)].

#### 3.3.4 Degree of disability: mRS and its subgroup analysis

**Figure 7:**
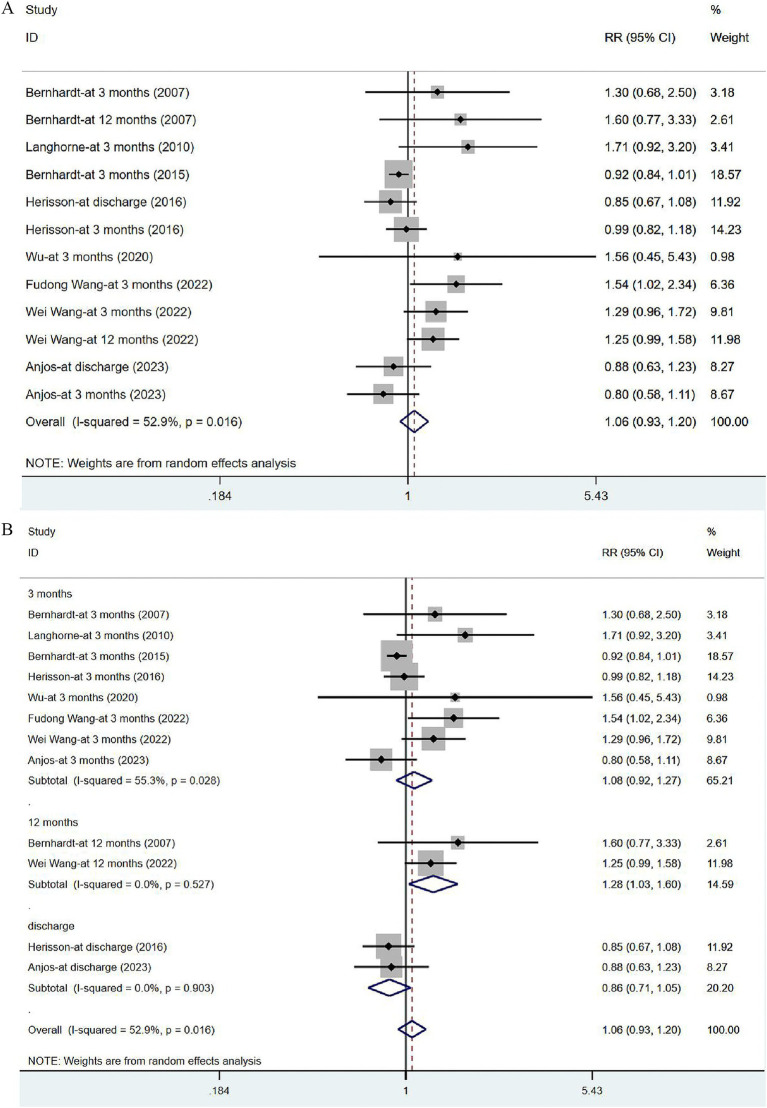
Forest plot of the disability degree. (A) Forest maps of meta-analysis of mRS. (B) Forest maps of subgroup analysis of mRS. (A) Comparison: the effect of VER on the mRS after stroke. Statistical method: Inverse-variance of the random effects model [relative risk (RR) and 95% confidence interval (CI)]. Very early rehabilitation (VER); modified Ranking Scale (mRS). (B) Comparison: the effect of VER on the mRS after stroke at different endpoints. Very early rehabilitation (VER); modified Ranking Scale (mRS); RR, risk ratio; 95% CI, 95% confidence interval.

Eight studies (17, 31, 33–35, 41, 43, 44) reported on mRS, with eight assessed at 3 months after the onset of stroke, two at 12 months and two at discharge. Given significant heterogeneity (*I*^2^ = 52.9%, *p* = 0.016), a random-effects model was utilized to analyze the mRS data, but no significant difference was noted [RR = 1.06, 95% CI (0.93, 1.20)] (Figure 7A). The sensitivity analysis showed a low sensitivity and stable results for this outcome (Supplementary Figure 1). Subgroup analysis indicated that the risk of serious disability in the VER group was lower than that in the control group [RR = 1.28, 95% CI (1.03, 1.60)] at 12 months, while there was no significant reduction in the risk of disability measured at discharge [RR = 0.86, 95% CI (0.71, 1.05)] and at 3 months after stroke [RR = 1.08, 95% CI (0.92, 1.27)] (Figure 7B).

#### 3.3.5 Daily activity ability: BI and its subgroup analysis

**Figure 8:**
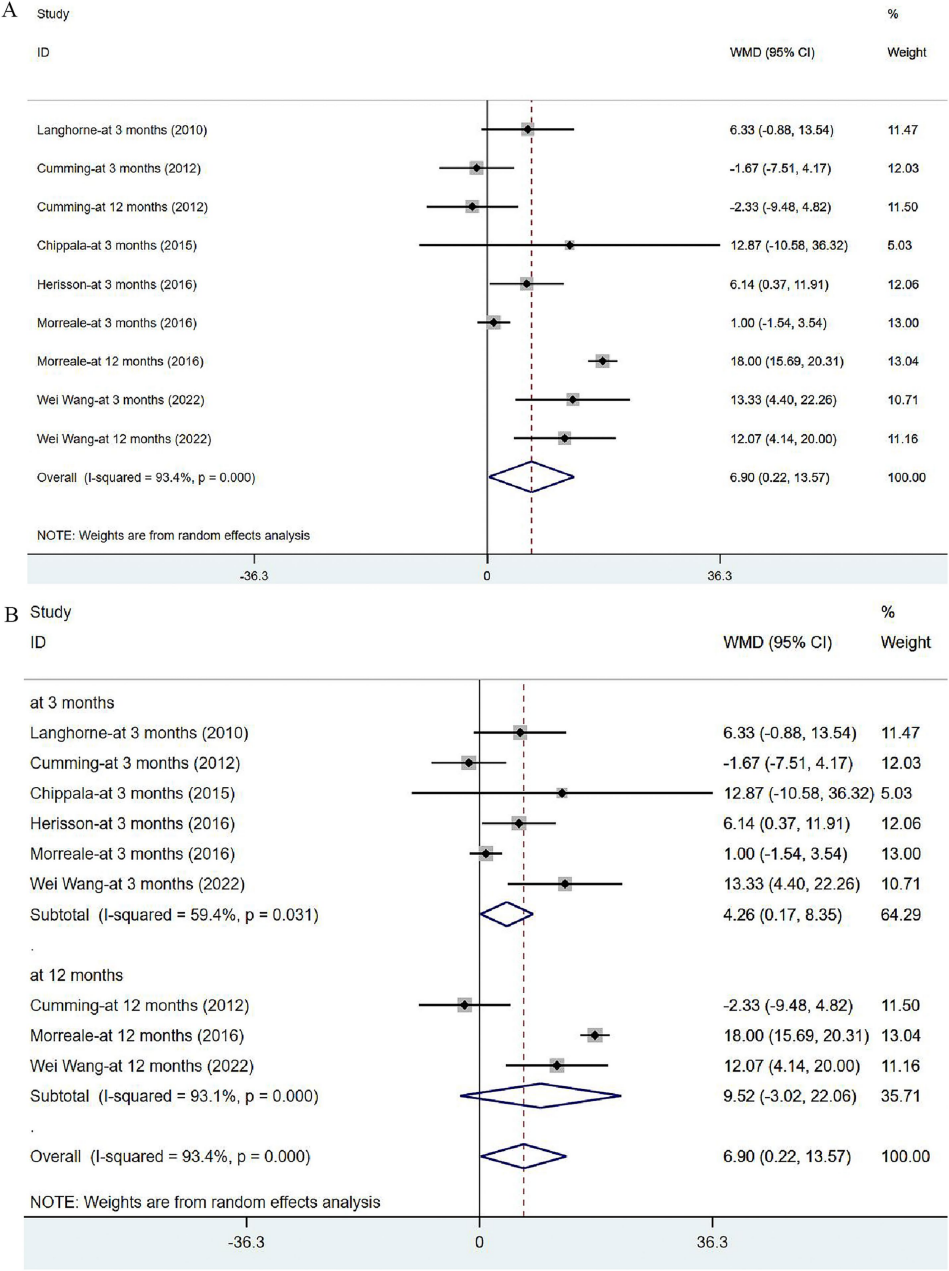
Forest plot of the daily living. (A) Forest maps of meta-analysis of BI. (B) Forest maps of subgroup analysis of BI. (A) Comparison: the effect of VER on BI. Statistical method: Inverse-variance of the random effects model [weighted mean difference (WMD) and 95% confidence interval (CI)]. Barthel Index (BI); very early rehabilitation (VER). (B) Comparison: the effect of VER on BI after stroke at different endpoints. Barthel Index (BI); very early rehabilitation (VER); weighted mean difference (WMD); 95% CI: 95% confidence interval.

Six articles (34, 36, 40–43) reported on BI in post-stroke patients. The studies were heterogeneous (*I*^2^ = 93.4%, *p* = 0.000). Meta-analysis was carried out with the random effects model, and the results suggested that VER could improve the BI of stroke patients [WMD = 6.90, 95% CI (0.22, 13.57)] (Figure 8A). None of these studies were found to influence the aggregated estimates in the sensitivity analysis (Supplementary Figure 2). In subgroup analysis, the improvement in BI remained significant at 3 months [WMD = 4.26, 95% CI (0.17, 8.35)], while no significant effect of VER on BI was noted at 12 months after stroke [WMD = 9.52, 95% CI (−3.02, 22.06)] (Figure 8B).

#### 3.3.6 Limb motor function: FMA

**Figure 9:**
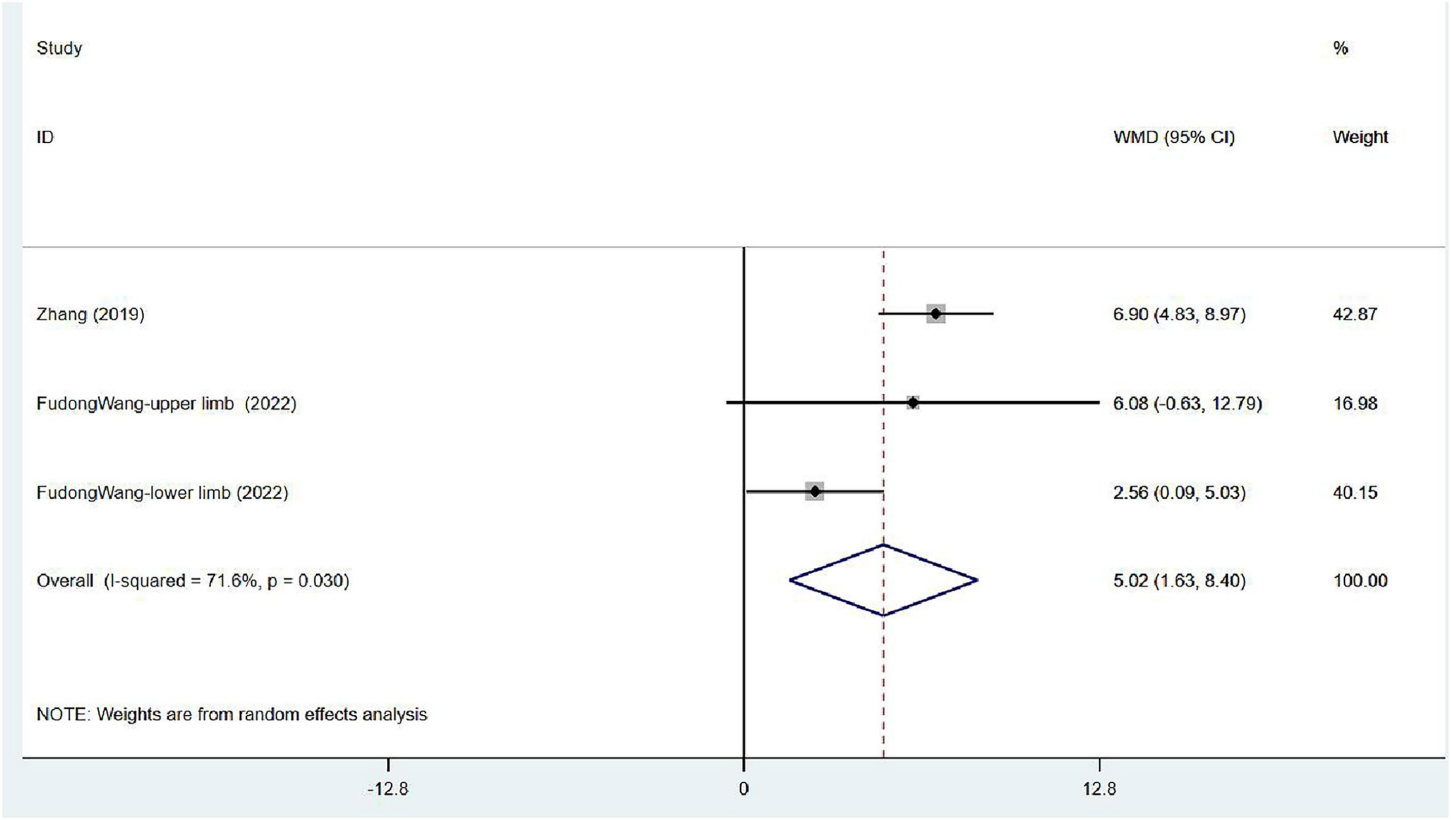
Forest plot of meta-analysis of limb motor function. Comparison: the effect of VER on FMA. Statistical method: Inverse-variance of the random effects model [weighted mean difference (WMD) and 95% confidence interval (CI)]. Fugl-Meyer assessment (FMA); very early rehabilitation (VER).

Two articles (32, 33) focused on FMA scores in patients after treatment. One assessed the overall FMA, and the other reported FMA scores for the upper and lower limbs separately. Moderate heterogeneity was noted (*I*^2^ = 71.6%, *p* = 0.030), and sensitivity analysis indicated low sensitivity (Supplementary Figure 3). The results of the random effects model indicated that VER contributed to the restoration of motor mobility [WMD = 5.02, 95% CI (1.63, 8.40)] (Figure 9).

#### 3.3.7 Proportion of walking 50 m unassisted

**Figure 10:**
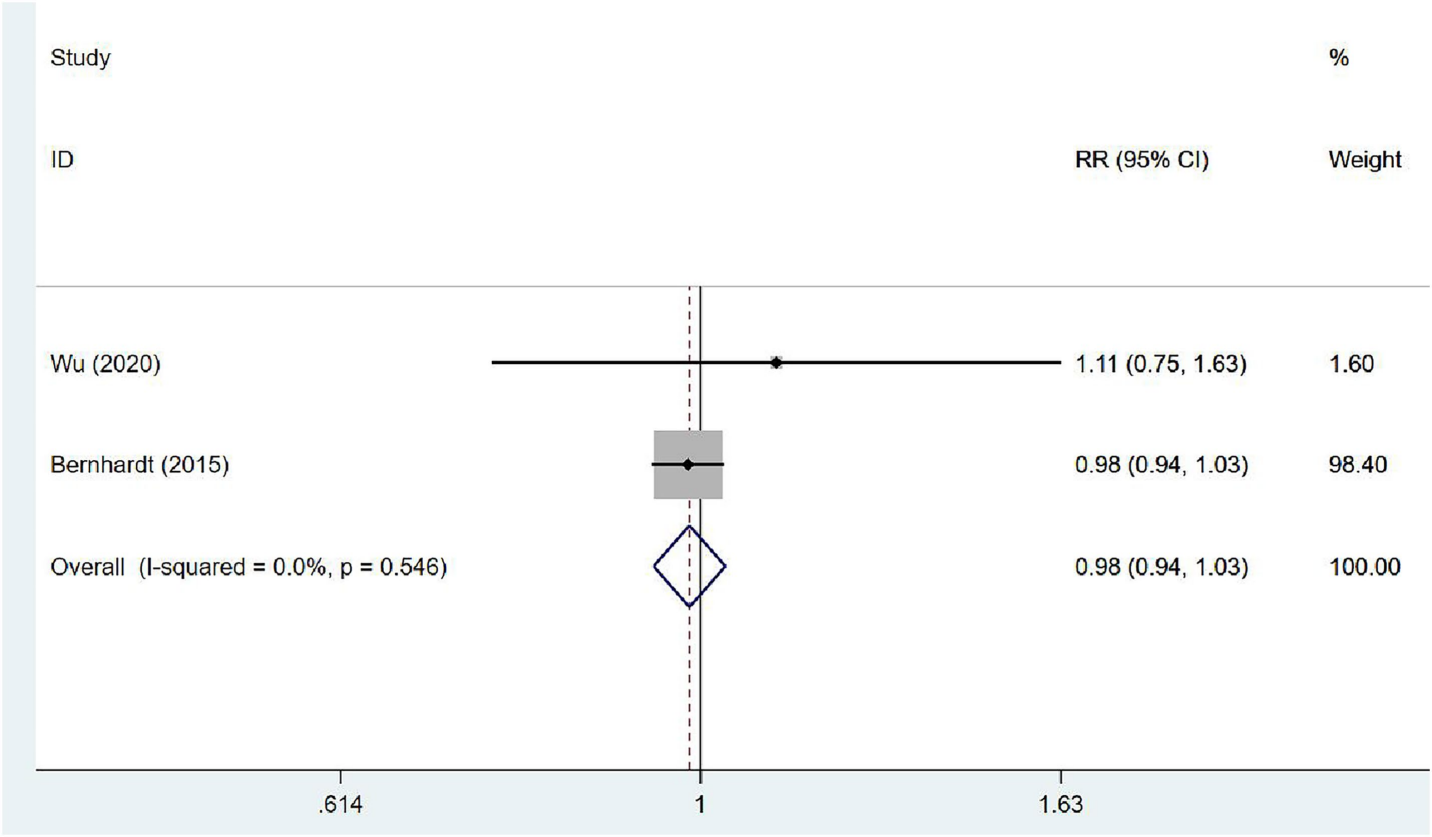
Forest plot of meta-analysis of the proportion of walking 50 m unassisted. Comparison: the effect of VER on the proportion of walking 50 m unassisted at 3 months after stroke. Statistical method: Inverse-variance of the fixed effects model [relative risk (RR) and 95% confidence interval (CI)]. very early rehabilitation (VER).

Two (31, 35) articles investigated the proportion of walking 50 m unassisted in patients at 3 months after stroke. The studies exhibited no heterogeneity (*I*^2^ = 0.0%, *p* = 0.546), and the fixed-effects model was used for meta-analysis [RR = 0.98, 95% CI (0.94, 1.03)], which demonstrated that VER cannot expedite the recovery of the ability to walk independently within 3 months (Figure 10).

#### 3.3.8 Publication bias

Publication bias was detected through funnel plots and Egger’s test for change in mortality, adverse events, NIHSS, mRS, BI, and FMA. Results indicated potential publication bias for mRS (*p* = 0.035) and no publication bias for mortality (*p* = 0.204), adverse events (*p* = 0.052), NIHSS (*p* = 0.174), BI (*p* = 0.558), and FMA (*p* = 0.947) (Supplementary Figures 4–9).

## 4 Discussion

This meta-analysis proved that VER could elevate the risk of death after stroke and stroke severity during hospitalization. However, it significantly lowered the risk of long-term complications or disability and had positive effects on improving daily living abilities and limb movement recovery after stroke.

Since the Swedish Consensus Conference on Stroke Care in the mid-1980s, VER, which comprises out-of-bed sitting, standing, and walking, has been recognized as an important part of unit care for acute stroke and incorporated into national guidelines around 1994 (45–47). Despite growing attention from guidelines and expert consensus on early rehabilitation, agreement was not reached regarding when (appropriate start time and duration) and how (intervention type, intensity, frequency, amount) to perform VER. Therefore, the safety and efficacy of VER in acute stroke patients remains a concern for clinicians.

A Cochrane review revealed a trend toward increased mortality at 3 months in the most relevant trials, although the difference was not significant between VER and delayed or lower-dose mobilization (11). Sundseth et al. noted a higher proportion of deaths at 3 months in the VER group mobilized within 24 h of admission in comparison to the control group mobilized between 24 and 48 h, but the difference was not significant though stroke severity was adjusted (48). The result of our meta-analysis showed that VER raised the risk of death in patients [RR = 1.27, 95% CI (1.00, 1.61)]. An important distinction between this study and others was the variation in assessment times. Among the eligible studies, one study considered 14-day mortality while others focused on mortality over 3 months. It is essential to exercise caution in concluding that VER-related mortality was more likely to occur shortly after stroke, as only one study has examined the 14-day mortality. Nonetheless, given the large sample size from the study included in the analysis, we cannot disregard that the observed 27% mortality rate may cause fatal harm to AIS population receiving VER treatment. Notably, the study indicated that patients aged over 80 or those with intracranial hemorrhage exhibited a higher mortality rate (39). During the progression of AIS, although the majority of patients experience gradual recovery, a significant number of patients have not substantially recovered or their conditions even deteriorated during the subsequent 24–72 h (49). This period was marked by considerable clinical uncertainty (45). Stroke-related events, including stroke progression or recurrence, are the most common causes of death within 14 days (39), and likely attributed to early neurological deterioration predominantly arising from intracranial hemorrhage and vasogenic edema (50, 51). The subgroup analysis of NIHSS in the present analysis revealed a statistically significant difference in NIHSS assessed at discharge between the two groups [WMD = 0.81, 95% CI (0.01, 1.61)], but no difference was observed at 3 months. This finding may offer insight into the elevated mortality noted in the VER group. Early neurological deterioration within 24 h of AIS was associated with increased mortality (50), which may be helpful for identifying predictors of death in patients undergoing early rehabilitation. Proximal arterial occlusion (52, 53), failure of recanalization and insufficient cerebral hemodynamic reserve (54) found through imaging were demonstrated to be correlated with deterioration. Moreover, severe white matter hyperintensities might be linked to poor prognosis after AIS due to impaired brain microcirculation (55). Elevated adrenomedullin has been considered as an independent predictor of AIS outcomes in recent years (56) and a novel plasma biomarker related to the increased mortality in AIS patients undergoing early rehabilitation (57), possibly due to the involvement of adrenomedullin receptor genes in vascular injury (58). Therefore, both image and plasma prediction tools can help clinicians identify VER patients at a high risk of death.

Premature out-of-bed movement and head position change within 24 or 48 h may induce unpredictable alterations in both intracranial and systemic hemodynamics (14, 59), and accelerate the deterioration, especially after 48 h of the onset of symptoms when the infarction edema reached its peak, which necessitates restricted head positioning (16). There were conflicting reports regarding the outcomes of AIS patients receiving thrombolytic therapy. Alteplase thrombolysis was expected to cause dropped embolus and a new embolic event, and induce hemorrhagic transformation, but the incidence of neurological deterioration in patients who did not have thrombolytic therapy was higher than those receiving thrombolysis (50). One study suggested that early rehabilitation reduced the risk of death within 3 months in patients with large artery occlusive stroke receiving endovascular treatment (60), whereas another small-scale study, which did not address endovascular therapy, reported increased mortality in patients who underwent early upright exercise. In addition to reconstructing brain circulation, adaptation to the state of ischemia or hypoxia to activate angiogenesis and neuroprotection is also a complementary strategy for early rehabilitation to avoid adverse outcomes. Intermittent hypercapnic hypoxia, which contributed to neuroprotection (61), could be applied in rehabilitation in stroke treatment, but current studies have not evaluated its impact on the mortality (62). Tong et al. investigated the safety of remote ischemic conditioning followed by exercise in the Phase 1 clinical trial and no significant difference was found in a small number of samples (63). Wang et al. demonstrated in a rat model that exercise following remote ischemic conditioning led to elevated expression of mRNA and proteins associated with neuroplasticity and angiogenesis (64). These studies explore a novel and comprehensive rehabilitation strategy, and larger clinical trials are needed in the future.

Complications arising from stroke can be categorized into those associated with immobility and neurological adverse events of stroke progression and current stroke, and the latter can be fatal for severely ill patients. Unlike patients recovering from surgery or other medical conditions like heart failure, asthma, or gastrointestinal bleeding, individuals with AIS are at an elevated risk for falls and costly hospital-acquired complications such as fractures, pneumonia, skin pressure ulcers, lower limb venous thrombosis, and pulmonary embolism (65, 66). The prevention of these complications is crucial not only for improving individuals’ long-term quality of life but also for easing the societal healthcare burden. Physical exercise in the later stage plays a crucial role in maintaining health, aiding muscle recovery, and preventing the complications associated with prolonged bed rest (46, 48), but the optimal time for initiating rehabilitation in the acute phase of stroke remains uncertain. Furthermore, despite the expansion and construction of stroke units, there was insufficient evidence to determine whether the reduction in complications is owing to routine mobilization of standard care or early rehabilitation (67, 68). A prior meta-analysis revealed that VER did not increase the incidence of adverse events, which was consistent with the results in this study [RR = 0.89, 95% CI (0.79, 1.01)]. Subgroup analyses demonstrated that early rehabilitation lowered the incidence of complications in the late stage (at 3 and 12 months) after stroke [RR = 0.86, 95% CI (0.74, 0.99)], although the effect was not significant for the early period (during hospitalization or within 14 days) [RR = 0.99, 95% CI (0.79, 1.24)]. VER was demonstrated to have a lasting effect in reducing adverse events. A retrospective study showed that patients with severe disabilities were about 2.5 times more likely to develop complications than those with mild disabilities (69). In conclusion, the reduction of long-term complications after stroke can be attributed to the beneficial effects of early rehabilitation on recovery of physical activities, which can shorten the time of immobility. A very early rehabilitation trial (AVERT) demonstrated that the longer patients remained hospitalized, the greater their likelihood of experiencing mobility-related complications (70). Nevertheless, the causal association between hospital stay length and the occurrence of complications remains unraveled. It is hypothesized that early rehabilitation may mitigate post-stroke complications by shortening hospital stays. Due to limitations in the available data, complications were not categorized according to their mechanisms. Future research could clarify the correlation between early rehabilitation and immobility-related complications by systematically categorizing complications based on their underlying mechanisms.

Our findings proved the effectiveness of VER for the recovery of living ability and limb movement in stroke patients, mainly at three or 12 months in the subgroup analysis, and indicated that its effects are long-lasting. The mRS of 0–2 proportion and BI were employed to assess daily life ability and independence, which were indicative of the quality of life in older age. Previous systematical reviews have demonstrated that intensive rehabilitation or physiotherapy was beneficial to the improvement of physical performance (71, 72). The most rapid improvement was observed within the first 6 months after stroke (72, 73), which was similar to the results related to BI in the present analysis, and a significant increase was noted at 3 months after the stroke onset [WMD = 4.26, 95% CI (0.17, 8.35)]. Therefore, the rate of recovery of physical function after stroke is not uniform. In comparison to the previous meta-analysis, our study incorporated the FMA and the proportion of walking 50 m unassisted to evaluate limb mobility. Patients in the VER group exhibited a notable increase in FMA scores, which indicated a significant improvement in limb function recovery. However, it’s important to acknowledge the limited scope of our analysis, as it was based on only two studies with differing assessment methodologies—one evaluating total scores and the other focusing on upper and lower limb function separately. Given the observed heterogeneity, these results should be interpreted with caution. VER stimulates marrow stem cells to differentiate into endothelial progenitor cells (12), thereby introducing angiogenesis in ischemic areas by boosting VEGF secretion (74, 75) and interacting with the expression of inflammatory factors in circulation. VER-induced increase of IF-10 and inhibition of TNF-α expression are not only associated with endothelial progenitor cells mobilization, but also play a significant role in anti-inflammation and neuroprotection during the acute phase of stroke (12). Furthermore, the brain is highly plastic after stroke because synaptic connections are formed and removed through constant activity (68), which proved that the recovery of nerve and following muscle function hinged on physical training. Biernaskie et al. conducted rehabilitation treatments for rats with cerebral middle artery occlusion at different times, and found that the recovery effect declined with delay of the start time of rehabilitation (76). This suggests that stroke recovery, which is contingent on the process in which a number of synapses re-establish connections and transmit signals (66), does have a time window. Therefore, the identification of the optimal time for initiating VER should be based on this window. However, determining the ideal early rehabilitation time after stroke remains challenging. Tong et al. conducted a study in which the experimental group received rehabilitation within 24 h, while the control group received it within 24–48 h. Their findings indicated that rehabilitation initiated at 48 h after stroke was beneficial, whereas VER within 24 h did not produce favorable outcomes at 3 months (77). Research on rats undergoing early training within 24 h showed increased apoptotic cell death (78, 79), which leads to enlarged infarcts.

In terms of recovery related to ADLs after stroke, both cognitive and motor tasks, especially those involving postural balance and walking, must be considered. A cross-sectional observational study involving 163 community-based chronic stroke survivors revealed that over half of participants had somatosensory impairments in the lower limb, especially in the distal regions, which was strongly correlated with the risk of falling (80). Proprioceptive training, which focuses on the body’s ability to recognize joint position and body movement, could better mobilize patients’ autonomy and cognitive function than traditional limb motor rehabilitation (81). Task-oriented exercises, with its increasing cognitive load, aided participants in real-world activities (82), and exhibited beneficial effects on proprioception, impaired gait, and spasticity (83). Constant sensory input of the limbs stimulates proprioception and decreases muscle stiffness (83). Published studies demonstrated that proprioceptive training, and dual-task exercises (cognitive and motor tasks) could significantly improve both balance and autonomy (81, 84, 85). However, it should also be noted that in the very early stage of AIS, the dual-task training on patients with limb weakness may lead to safety problems such as falls and sprains. In addition, speech training was proved to be crucial for addressing communication impairments (dysarthria) in clinical practice and was an important part of post-stroke rehabilitation, because people with dysarthria would have no social participation and be ignored in communication (86). Therefore, early rehabilitation after stroke should be comprehensive and cover motor, cognition, proprioception and speech, thereby offering a more comfortable experience for the post-stroke population. A personalized and enriched rehabilitation program can both improve adherence and mitigate the negative emotion caused by hemiplegia and dysarthria.

The present meta-analysis has several limitations. First, the strict criteria adopted to define early rehabilitation time led to a reduced number of eligible studies, so the overall sample size was small. Moreover, the robustness of the subgroup analysis results may be compromised due to the limited number of studies available. Second, many studies were excluded from the quantitative synthesis due to different data types of outcome indicators between studies, which may have resulted in bias. Third, the included studies varied in the duration, dose, intensity, and frequency of rehabilitation treatments, and the final results might have been influenced by multiple variables. Fourth, in some studies, out-of-bed activities may be initiated too early in the control population, so there existed confusion about whether the result was influenced by standard care intervention. Fifth, in this research, a 24-h time threshold was not set for comparing the prognosis of VER initiated within 24 h versus 24–48 h. Lastly, stroke type and stroke treatment in the acute phase (intravenous thrombolysis, embolectomy, usual medication) were not systematically categorized due to limited studies.

## 5 Conclusion

In conclusion, VER could improve ADLs, lower the incidence of long-term complications in stroke survivors, and maintain such effectiveness. However, premature and overly intensive rehabilitation may elevate the risk of death in AIS patients in the acute phase. Statistics revealed that a growing number of patients with severe symptoms tended to receive rehabilitation in post-stroke specialized units, which is driven by demographic trend toward an aging population (69). Therefore, achieving both safety and efficacy in VER necessitates a comprehensive consideration of multiple factors and the development of tailored strategies. Attention should be paid not only to the initiation time of rehabilitation treatment in the acute phase of AIS, but also to the cultivation of physical exercise habits after discharge.

## Data Availability

The original contributions presented in the study are included in the article/Supplementary material, further inquiries can be directed to the corresponding author.
