# Supplementary material for: Efficacy and safety of very early rehabilitation for acute ischemic stroke: a systematic review and meta-analysis
Source: Front Neurol. 2024 Oct 22;15:1423517. doi: 10.3389/fneur.2024.1423517 (PMC11534803; doi:10.3389/fneur.2024.1423517)
Supplement: Supplementary file 1 [file Table_1.DOCX]

Supplementary Material

# Supplementary Data

**Supplementary Material 1.** Search terms and strategies.

**1. PubMed**

((("Ischemic Stroke"[Mesh]) OR (((((((((((((((((((((((((Ischemic Stroke[Title/Abstract]) OR (Ischemic Strokes[Title/Abstract])) OR (Stroke, Ischemic[Title/Abstract])) OR (Ischaemic Stroke[Title/Abstract])) OR (Ischaemic Strokes[Title/Abstract])) OR (Stroke, Ischaemic[Title/Abstract])) OR (Cryptogenic Ischemic Stroke[Title/Abstract])) OR (Cryptogenic Ischemic Strokes[Title/Abstract])) OR (Ischemic Stroke, Cryptogenic[Title/Abstract])) OR (Stroke, Cryptogenic Ischemic[Title/Abstract])) OR (Cryptogenic Stroke[Title/Abstract])) OR (Cryptogenic Strokes[Title/Abstract])) OR (Stroke, Cryptogenic[Title/Abstract])) OR (Cryptogenic Embolism Stroke[Title/Abstract])) OR (Cryptogenic Embolism Strokes[Title/Abstract])) OR (Embolism Stroke, Cryptogenic[Title/Abstract])) OR (Stroke, Cryptogenic Embolism[Title/Abstract])) OR (Wake-up Stroke[Title/Abstract])) OR (Stroke, Wake-up[Title/Abstract])) OR (Wake up Stroke[Title/Abstract])) OR (Wake-up Strokes[Title/Abstract])) OR (Acute Ischemic Stroke[Title/Abstract])) OR (Acute Ischemic Strokes[Title/Abstract])) OR (Ischemic Stroke, Acute[Title/Abstract])) OR (Stroke, Acute Ischemic[Title/Abstract]))) AND (("Early Ambulation"[Mesh]) OR (((((((((Early Ambulation[Title/Abstract]) OR (Accelerated Ambulation[Title/Abstract])) OR (Ambulation, Accelerated[Title/Abstract])) OR (Ambulation, Early[Title/Abstract])) OR (Early Mobilization[Title/Abstract])) OR (Mobilization, Early[Title/Abstract])) OR (Rehabilitation[Title/Abstract])) OR (Habilitation[Title/Abstract])) OR (Early[Title/Abstract])))) AND (randomized controlled trial[Publication Type] OR randomized[Title/Abstract] OR placebo[Title/Abstract])

**2. Embase**

| No. | Query | Results |
| --- | --- | --- |
| #43 | #27 AND #38 AND #42 | 854 |
| #42 | #39 OR #40 OR #41 | 877372 |
| #41 | 'double-blind':ab,ti | 228024 |
| #40 | 'placebo':ab,ti | 371691 |
| #39 | 'random':ab,ti | 445961 |
| #38 | #28 OR #29 OR #30 OR #31 OR #32 OR #33 OR #34 OR #35 OR #36 OR #37 | 2942963 |
| #37 | 'early':ab,ti | 2600051 |
| #36 | 'habilitation':ab,ti | 1804 |
| #35 | 'rehabilitation':ab,ti | 293587 |
| #34 | 'mobilization, early':ab,ti | 103 |
| #33 | 'early mobilization':ab,ti | 5375 |
| #32 | 'ambulation, early':ab,ti | 46 |
| #31 | 'ambulation, accelerated':ab,ti | 0 |
| #30 | 'accelerated ambulation':ab,ti | 10 |
| #29 | 'mobilization':ab,ti | 79084 |
| #28 | 'mobilization'/exp | 40212 |
| #27 | #1 OR #2 OR #3 OR #4 OR #5 OR #6 OR #7 OR #8 OR #9 OR #10 OR #11 OR #12 OR #13 OR #14 OR #15 OR #16 OR #17 OR #18 OR #19 OR #20 OR #21 OR #22 OR #23 OR #24 OR #25 OR #26 | 138225 |
| #26 | 'stroke, acute ischemic':ab,ti | 52 |
| #25 | 'ischemic stroke, acute':ab,ti | 189 |
| #24 | 'acute ischemic strokes':ab,ti | 860 |
| #23 | 'acute ischemic stroke':ab,ti | 38578 |
| #22 | 'wake-up strokes':ab,ti | 185 |
| #21 | 'wake up stroke':ab,ti | 495 |
| #20 | 'stroke, wake-up':ab,ti | 22 |
| #19 | 'wake-up stroke':ab,ti | 495 |
| #18 | 'stroke, cryptogenic embolism':ab,ti | 0 |
| #17 | 'embolism stroke, cryptogenic':ab,ti | 0 |
| #16 | 'cryptogenic embolism strokes':ab,ti | 0 |
| #15 | 'cryptogenic embolism stroke':ab,ti | 1 |
| #14 | 'stroke, cryptogenic':ab,ti | 25 |
| #13 | 'cryptogenic strokes':ab,ti | 540 |
| #12 | 'cryptogenic stroke':ab,ti | 3666 |
| #11 | 'stroke, cryptogenic ischemic':ab,ti | 0 |
| #10 | 'ischemic stroke, cryptogenic':ab,ti | 9 |
| #9 | 'cryptogenic ischemic strokes':ab,ti | 43 |
| #8 | 'cryptogenic ischemic stroke':ab,ti | 404 |
| #7 | 'stroke, ischaemic':ab,ti | 278 |
| #6 | 'ischemic stroke':ab,ti | 109849 |
| #5 | 'ischaemic strokes':ab,ti | 1323 |
| #4 | 'ischaemic stroke':ab,ti | 14881 |
| #3 | 'stroke, ischemic':ab,ti | 1236 |
| #2 | 'ischemic strokes':ab,ti | 7858 |
| #1 | 'ischemic stroke'/exp | 31977 |

**3. Cochrane Library**

| ID | Search | Hits |
| --- | --- | --- |
| #1 | MeSH descriptor: [Ischemic Stroke] explode all trees | 1063 |
| #2 | (Ischemic Stroke):ti,ab,kw OR (Ischemic Strokes):ti,ab,kw OR (Stroke, Ischemic):ti,ab,kw OR (Ischaemic Stroke):ti,ab,kw OR (Ischaemic Strokes):ti,ab,kw | 19330 |
| #3 | (Stroke, Ischaemic):ti,ab,kw OR (Cryptogenic Ischemic Stroke):ti,ab,kw OR (Cryptogenic Ischemic Strokes):ti,ab,kw OR (Ischemic Stroke, Cryptogenic):ti,ab,kw OR (Stroke, Cryptogenic Ischemic):ti,ab,kw | 19192 |
| #4 | (Cryptogenic Stroke):ti,ab,kw OR (Cryptogenic Strokes):ti,ab,kw OR (Stroke, Cryptogenic):ti,ab,kw OR (Cryptogenic Embolism Stroke):ti,ab,kw OR (Cryptogenic Embolism Strokes):ti,ab,kw | 272 |
| #5 | (Embolism Stroke, Cryptogenic):ti,ab,kw OR (Stroke, Cryptogenic Embolism):ti,ab,kw OR (Wake-up Stroke):ti,ab,kw OR (Stroke, Wake-up):ti,ab,kw OR (Wake up Stroke):ti,ab,kw | 320 |
| #6 | (Wake-up Strokes):ti,ab,kw OR (Acute Ischemic Stroke):ti,ab,kw OR (Acute Ischemic Strokes):ti,ab,kw OR (Ischemic Stroke, Acute):ti,ab,kw OR (Stroke, Acute Ischemic):ti,ab,kw | 9632 |
| #7 | #1 OR #2 OR #3 OR #4 OR #5 OR #6 | 19588 |
| #8 | MeSH descriptor: [Early Ambulation] explode all trees | 458 |
| #9 | (Early Ambulation):ti,ab,kw OR (Accelerated Ambulation):ti,ab,kw OR (Ambulation, Accelerated):ti,ab,kw OR (Ambulation, Early):ti,ab,kw OR (Early Mobilization):ti,ab,kw | 3629 |
| #10 | (Mobilization, Early):ti,ab,kw OR (Rehabilitation):ti,ab,kw OR (Habilitation):ti,ab,kw OR (Early):ti,ab,kw | 207364 |
| #11 | #8 OR #9 OR #10 | 207372 |
| #12 | #7 AND #11 | 3832 (3779 Trials and 53 reviews) |

**4. Web of Science**

| # | Search Query | Results |
| --- | --- | --- |
| 1 | "TS=(Ischemic Stroke) OR TS=(Ischemic Strokes) OR TS=(Stroke, Ischemic) OR TS=(Ischaemic Stroke) OR TS=(Ischaemic Strokes) OR TS=(Stroke, Ischaemic) OR TS=(Cryptogenic Ischemic Stroke) OR TS=(Cryptogenic Ischemic Strokes) OR TS=(Ischemic Stroke, Cryptogenic) OR TS=(Stroke, Cryptogenic Ischemic) OR TS=(Cryptogenic Stroke) OR TS=(Cryptogenic Strokes) OR TS=(Stroke, Cryptogenic) OR TS=(Cryptogenic Embolism Stroke) OR TS=(Cryptogenic Embolism Strokes) OR TS=(Embolism Stroke, Cryptogenic) OR TS=(Stroke, Cryptogenic Embolism) OR TS=(Wake-up Stroke) OR TS=(Stroke, Wake-up) OR TS=(Wake up Stroke) OR TS=(Wake-up Strokes) OR TS=(Acute Ischemic Stroke) OR TS=(Acute Ischemic Strokes) OR TS=(Ischemic Stroke, Acute) OR TS=(Stroke, Acute Ischemic)" | 147450 |
| 2 | "TS=(Early Ambulation) OR TS=(Accelerated Ambulation) OR TS=(Ambulation, Accelerated) OR TS=(Ambulation, Early) OR TS=(Early Mobilization) OR TS=(Mobilization, Early) OR TS=(Rehabilitation) OR TS=(Habilitation) OR TS=(Early)" | 3406194 |
| 3 | "TS=(randomized controlled trial) OR TS=(randomized) OR TS=(placebo) OR TS=(random) OR TS=(double-blind)" | 2214336 |
| 4 | "#3 AND #2 AND #1" | 3560 |

# Supplementary Figures

## Supplementary Figures


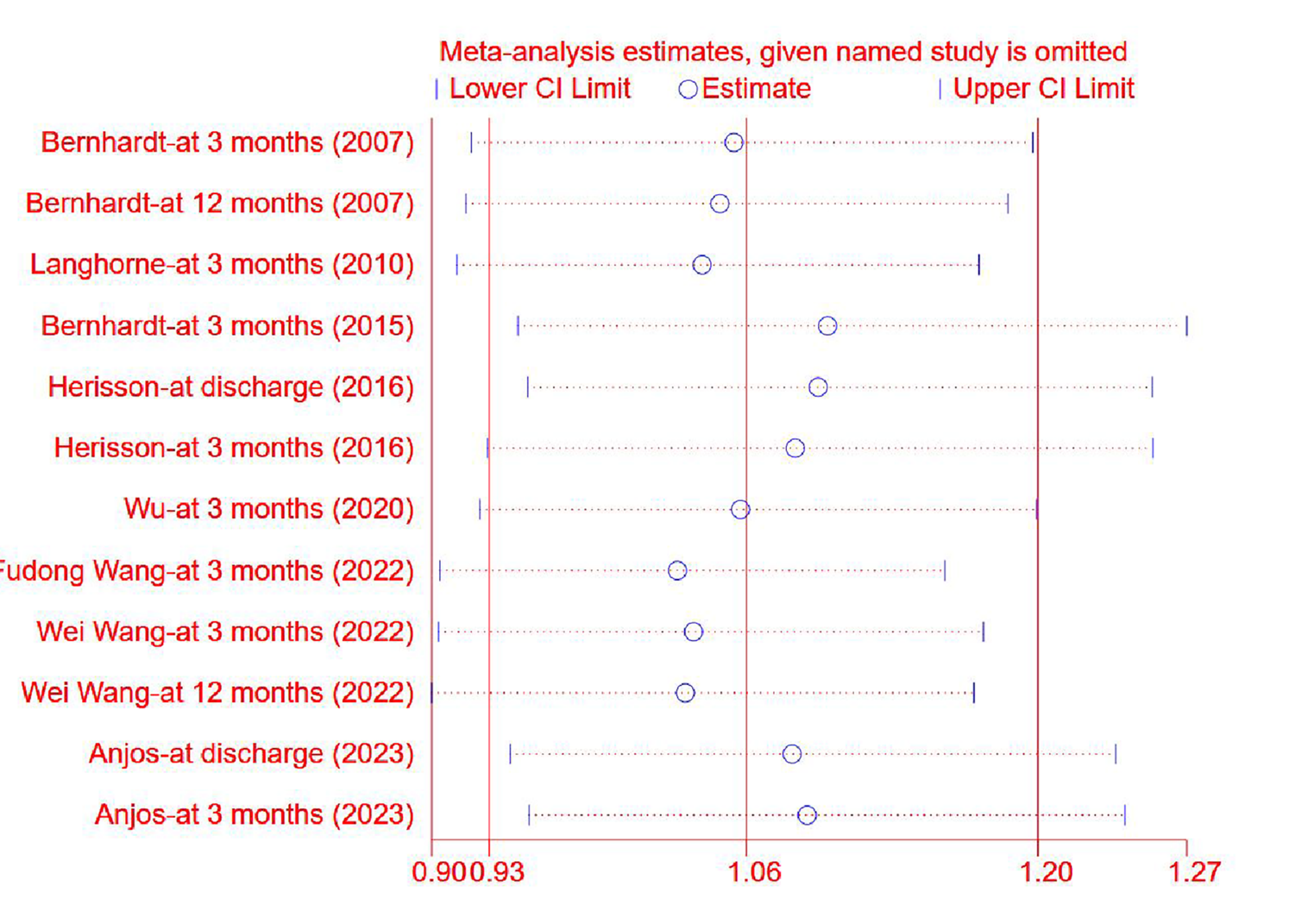


**Supplementary Figure 1.** Sensitivity analysis of mRS.

**Abbreviations:** mRS, modified Ranking Scale; CI, confidence interval.


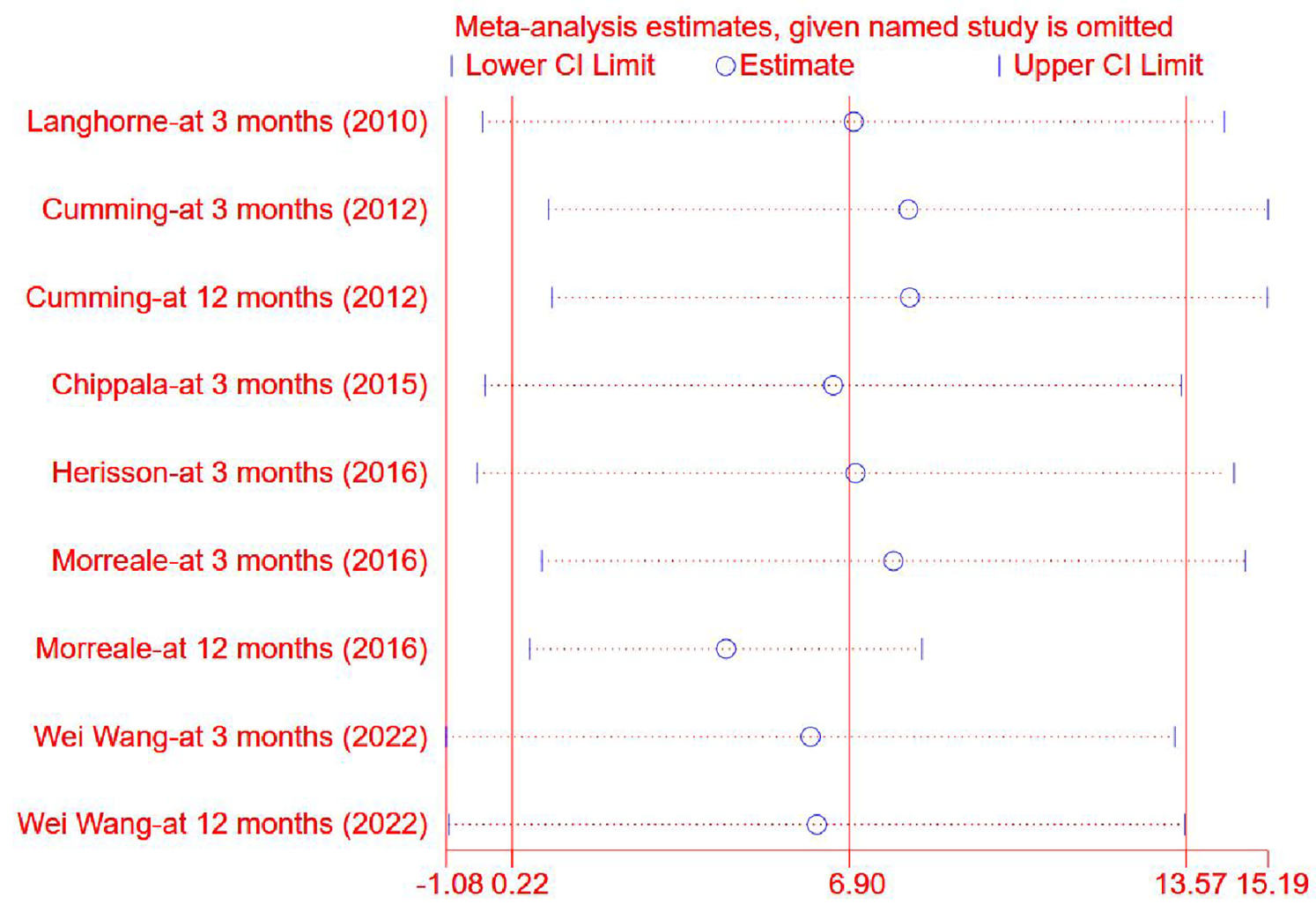


**Supplementary Figure 2.** Sensitivity analysis of BI.

**Abbreviations:** BI, Barthel Index; CI, confidence interval.


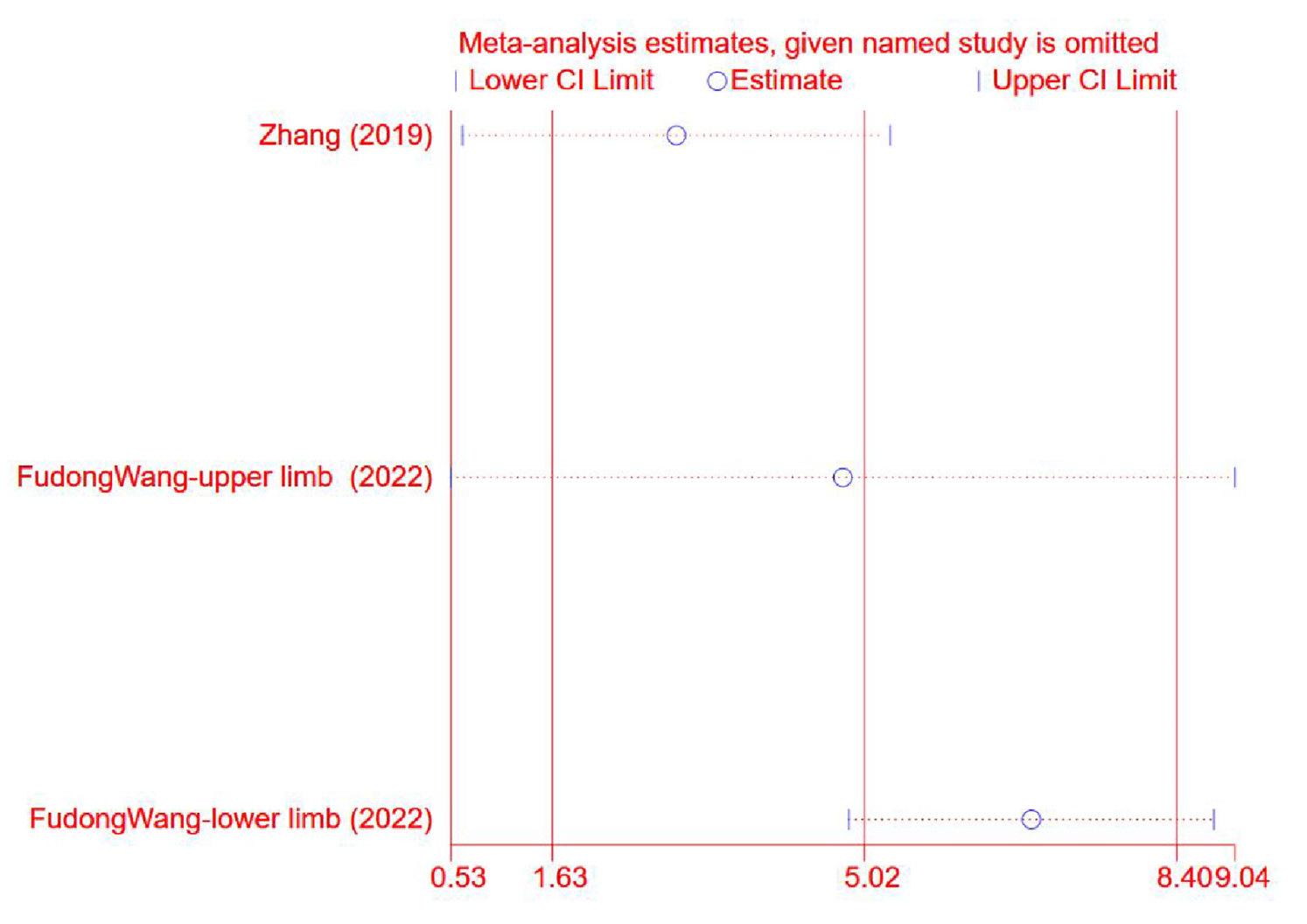


**Supplementary Figure 3.** Sensitivity analysis of FMA.

**Abbreviations:** FMA, Fugl–Meyer assessment; CI, confidence interval.


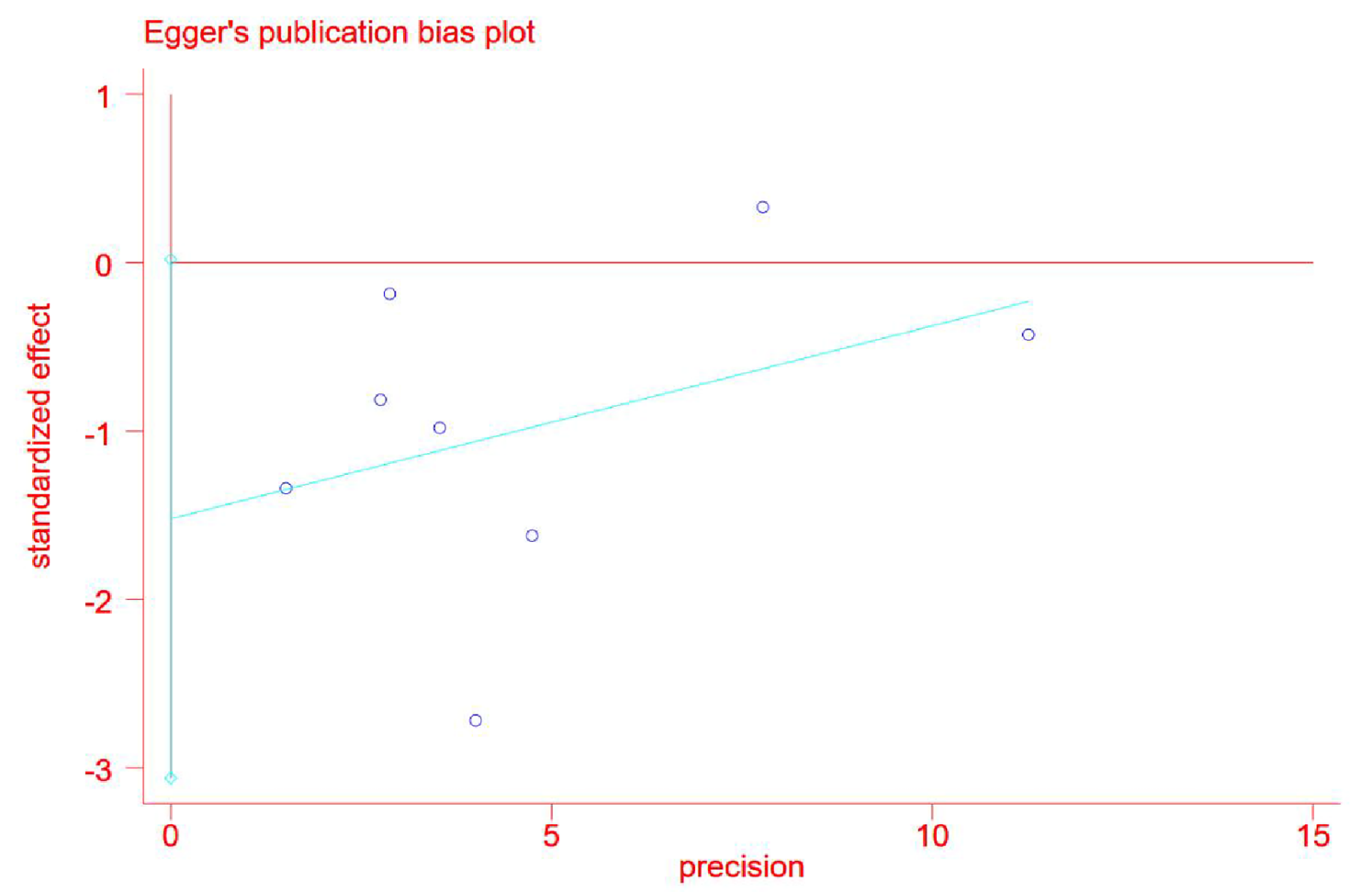


**Supplementary Figure 4.** Egger’s publication bias plot of mortality.


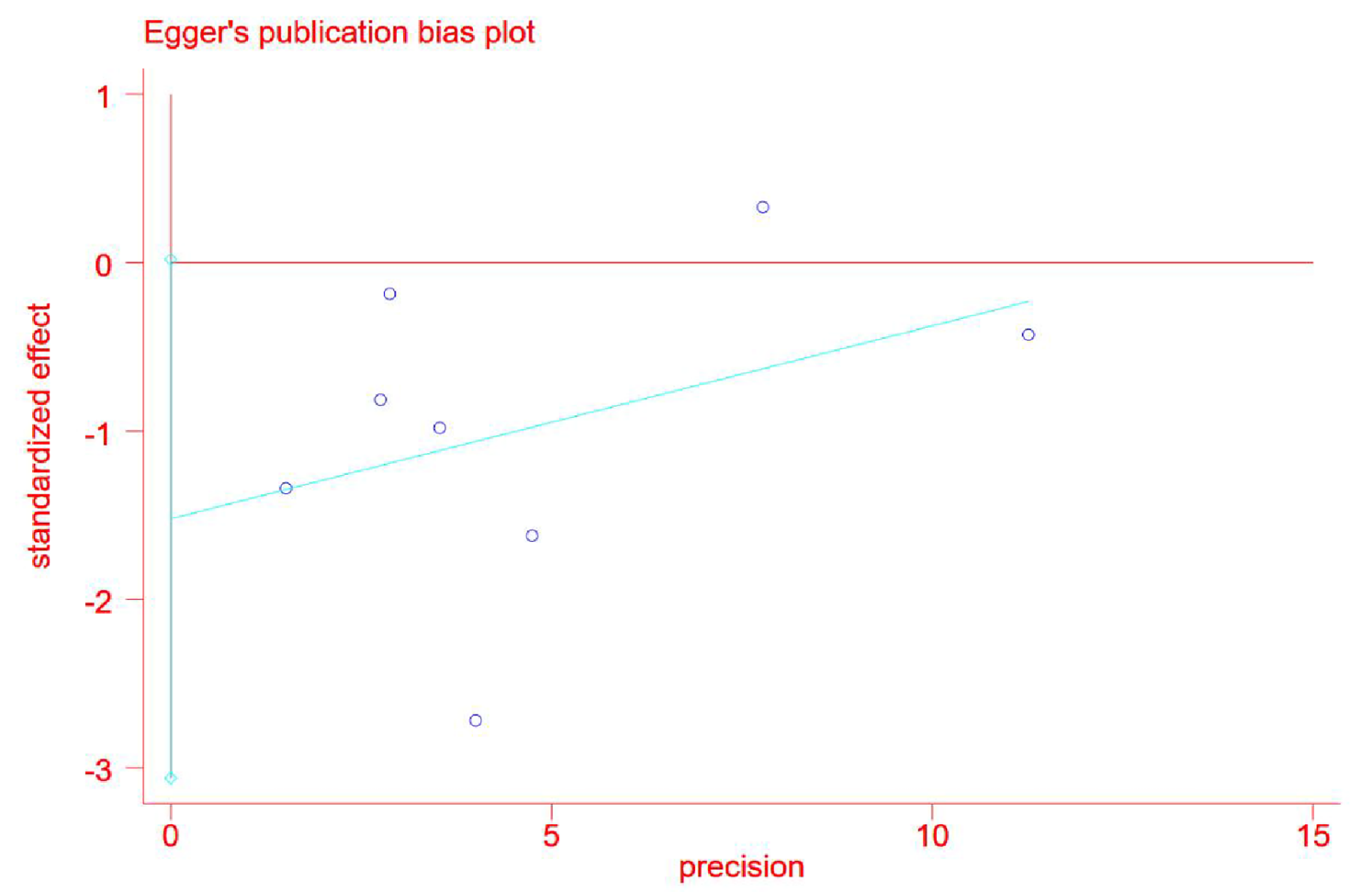


**Supplementary Figure 5.** Egger’s publication bias plot of adverse events.


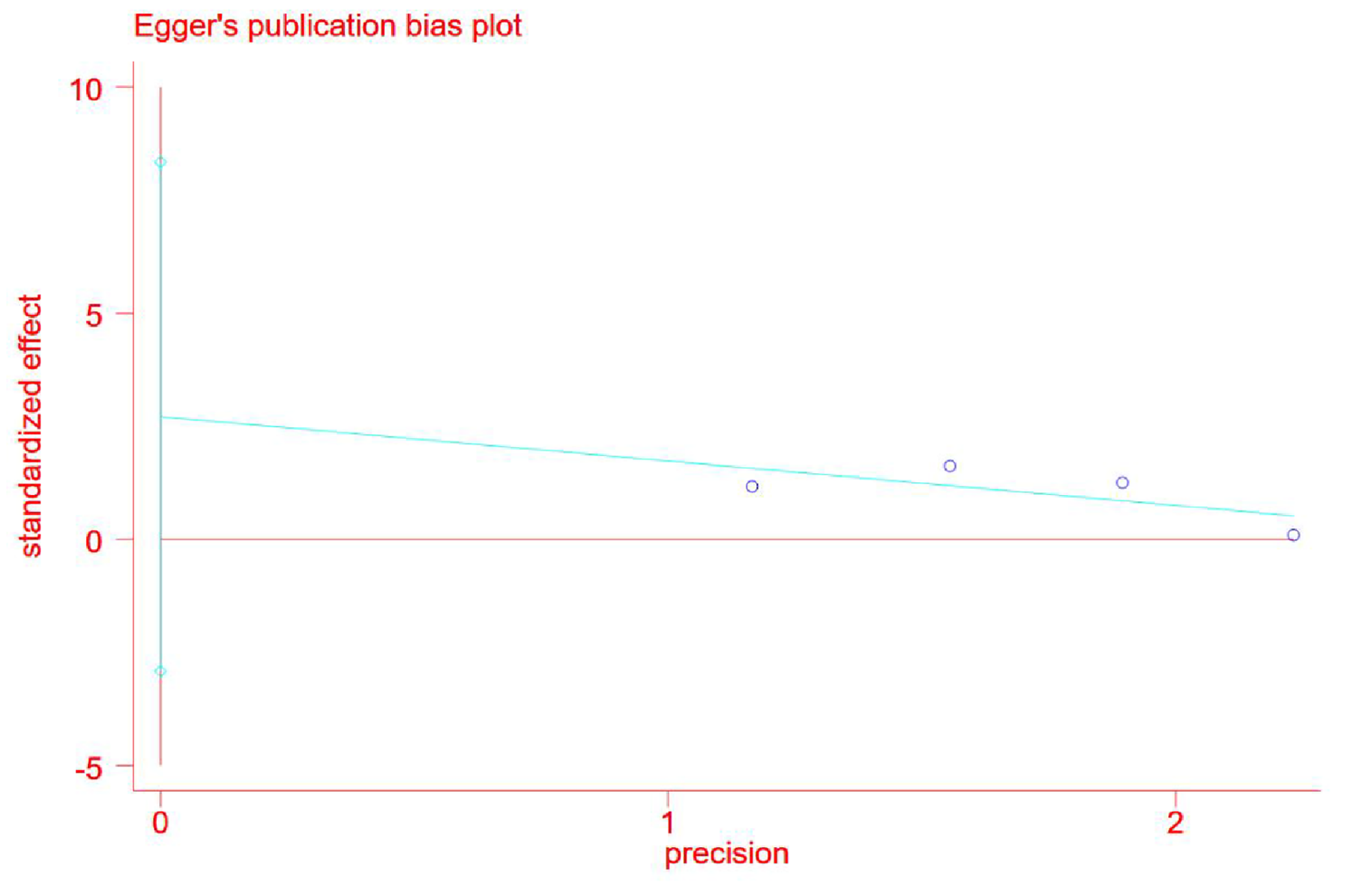


**Supplementary Figure 6.** Egger’s publication bias plot of NIHSS.

**Abbreviation:** NIHSS, National Institutes of Health Stroke Scale.


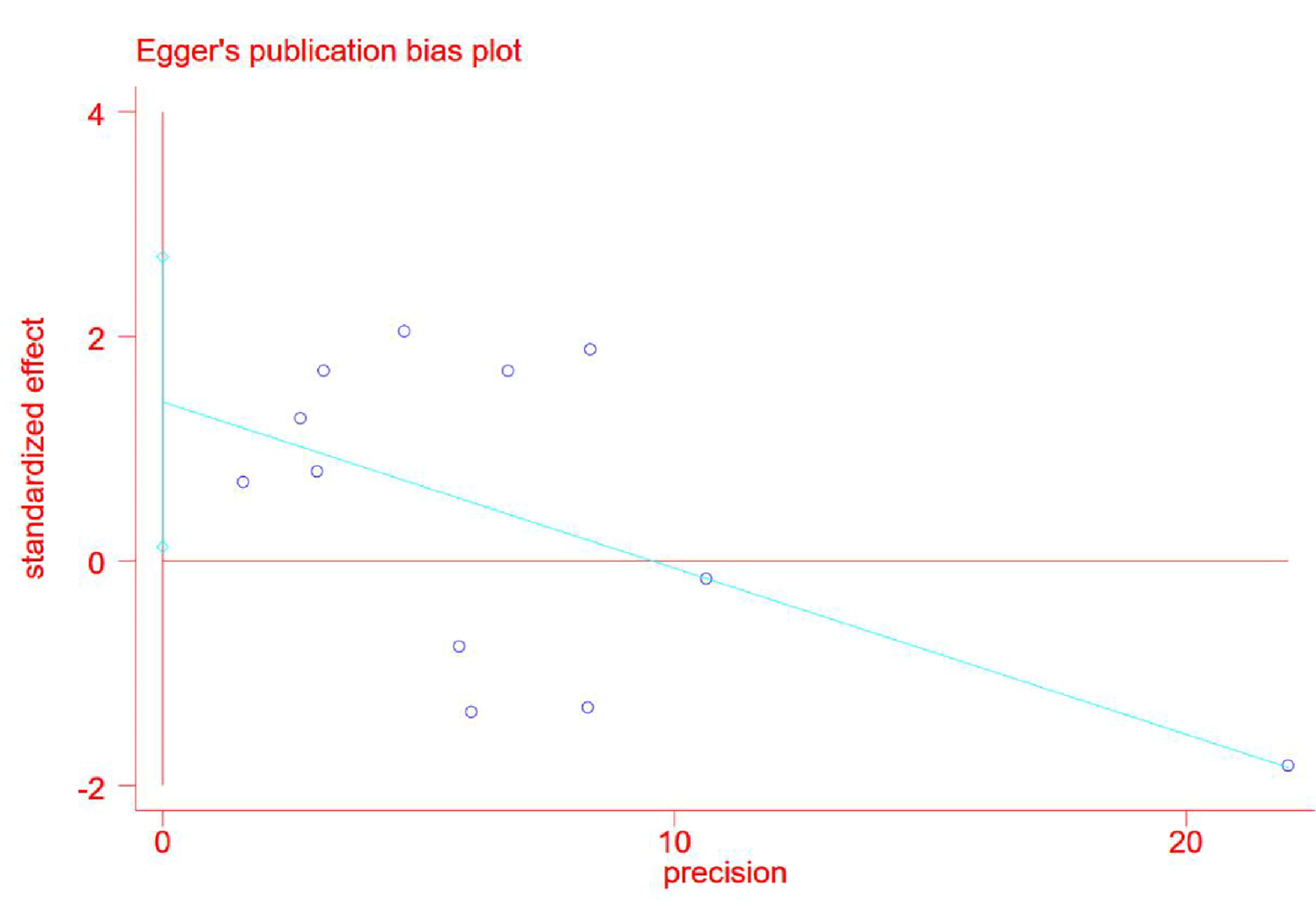


**Supplementary Figure 7.** Egger’s publication bias plot of mRS.

**Abbreviation:** mRS, modified Ranking Scale.


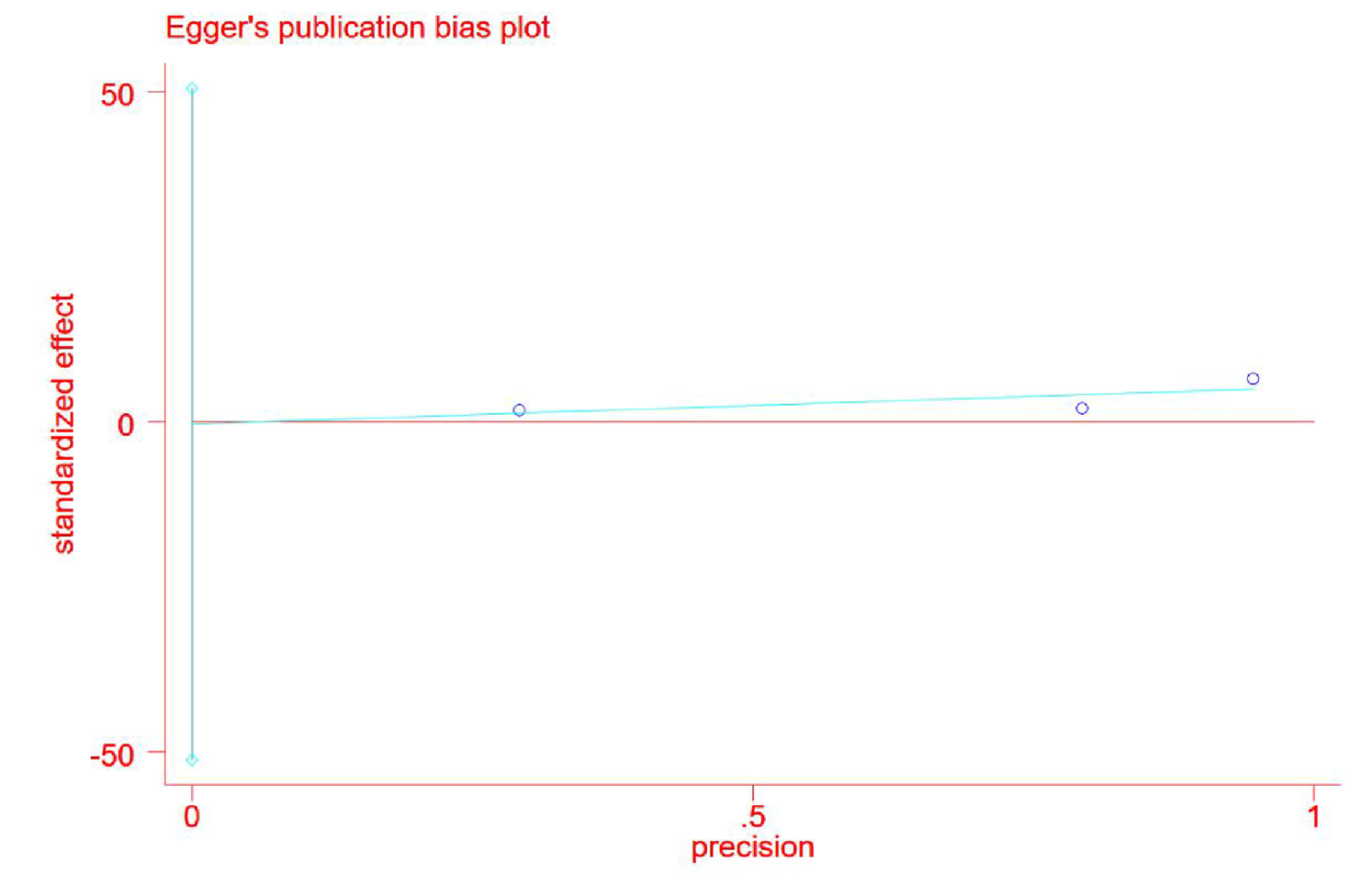


**Supplementary Figure 8.** Egger’s publication bias plot of BI.

**Abbreviation:** BI, Barthel Index.


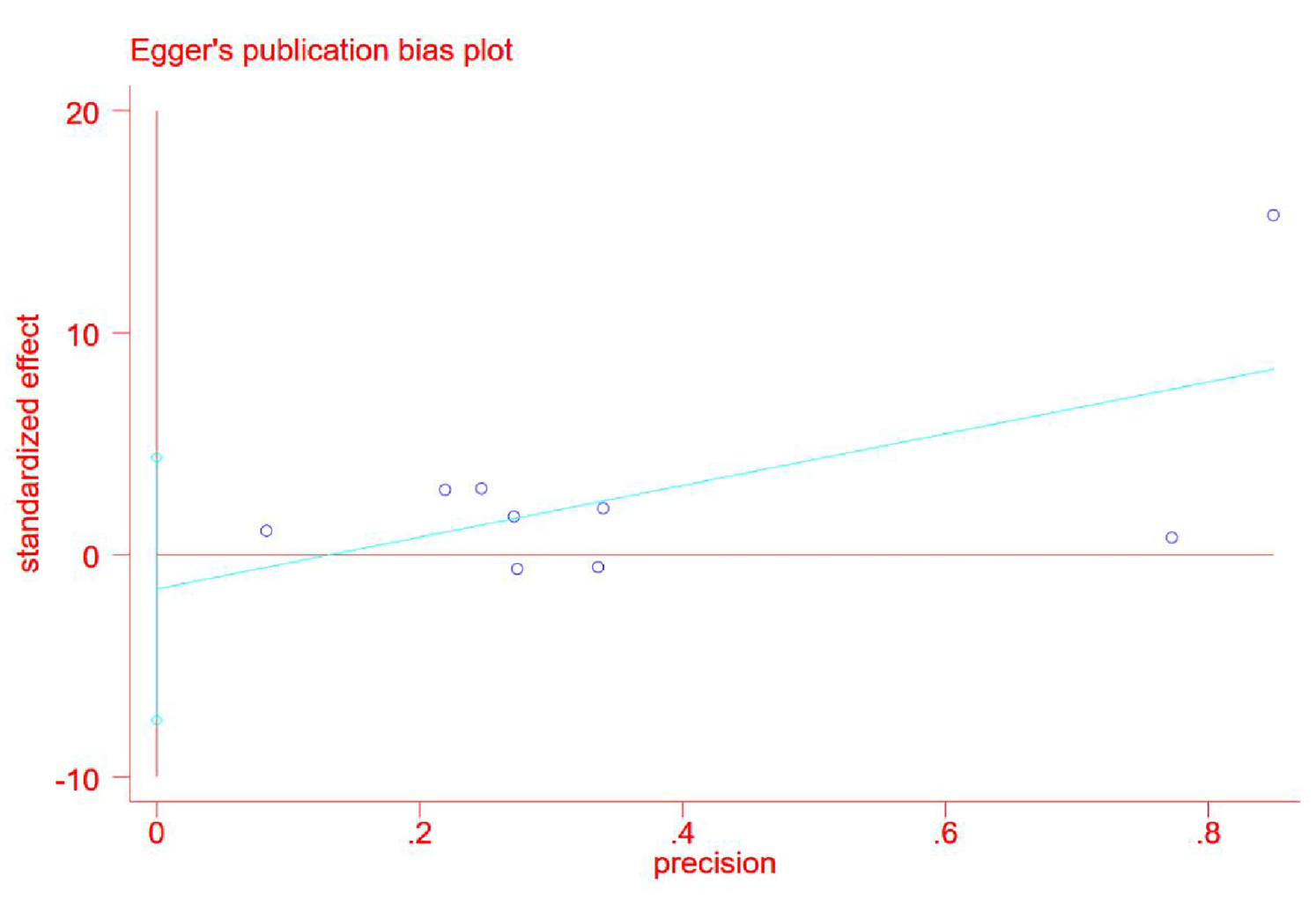


**Supplementary Figure 9.** Egger’s publication bias plot of FMA.

**Abbreviation:** FMA, Fuel-Meyer assessment.
